# Measurement of Outflow Facility Using *iPerfusion*

**DOI:** 10.1371/journal.pone.0150694

**Published:** 2016-03-07

**Authors:** Joseph M. Sherwood, Ester Reina-Torres, Jacques A. Bertrand, Barnaby Rowe, Darryl R. Overby

**Affiliations:** 1 Department of Bioengineering, Faculty of Engineering, Imperial College London, London, United Kingdom; 2 Department of Physics and Astronomy, Faculty of Mathematical and Physical Sciences, University College London, London, United Kingdom; Casey Eye Institute, UNITED STATES

## Abstract

Elevated intraocular pressure (IOP) is the predominant risk factor for glaucoma, and reducing IOP is the only successful strategy to prevent further glaucomatous vision loss. IOP is determined by the balance between the rates of aqueous humour secretion and outflow, and a pathological reduction in the hydraulic conductance of outflow, known as outflow facility, is responsible for IOP elevation in glaucoma. Mouse models are often used to investigate the mechanisms controlling outflow facility, but the diminutive size of the mouse eye makes measurement of outflow technically challenging. In this study, we present a new approach to measure and analyse outflow facility using *iPerfusion*^™^, which incorporates an actuated pressure reservoir, thermal flow sensor, differential pressure measurement and an automated computerised interface. In enucleated eyes from C57BL/6J mice, the flow-pressure relationship is highly non-linear and is well represented by an empirical power law model that describes the pressure dependence of outflow facility. At zero pressure, the measured flow is indistinguishable from zero, confirming the absence of any significant pressure independent flow in enucleated eyes. Comparison with the commonly used 2-parameter linear outflow model reveals that inappropriate application of a linear fit to a non-linear flow-pressure relationship introduces considerable errors in the estimation of outflow facility and leads to the false impression of pressure-independent outflow. Data from a population of enucleated eyes from C57BL/6J mice show that outflow facility is best described by a lognormal distribution, with 6-fold variability between individuals, but with relatively tight correlation of facility between fellow eyes. *iPerfusion* represents a platform technology to accurately and robustly characterise the flow-pressure relationship in enucleated mouse eyes for the purpose of glaucoma research and with minor modifications, may be applied *in vivo* to mice, as well as to eyes from other species or different biofluidic systems.

## Introduction

Intraocular pressure (IOP) is regulated by the balance between the secretion of aqueous humour (AH) and its outflow across the hydrodynamic resistance of the conventional outflow pathway. An increase in outflow resistance can lead to ocular hypertension, which is a major risk factor in glaucoma; a disease for which a reduction of IOP is the sole therapeutic target for sight preservation [1–3]. The physiology of AH outflow is complex, involving flow through both the trabecular meshwork and Schlemm’s canal (conventional outflow pathway) and the ciliary muscle, choroid and sclera (unconventional outflow pathway). Although the conventional pathway is the predominant outflow route in humans and increased resistance of this pathway is largely responsible for the elevated IOP observed in glaucoma [4], current medical treatments generally either promote unconventional outflow or reduce AH secretion [5, 6]. An increasing amount of research is therefore being aimed at developing treatments that target the root cause of ocular hypertension, namely increased resistance of the conventional outflow pathway [7, 8].

Mouse eyes are becoming an increasingly popular model for investigating the mechanisms of outflow and the hydrodynamic conductance of the conventional outflow pathway, known as outflow facility (the reciprocal of hydrodynamic resistance). Recent studies have shown that this pathway is anatomically similar in mice to that in human eyes [9, 10] and exhibits a comparable pharmacological response to drugs that both increase and decrease outflow facility [11–14]. In addition, the ability to genetically manipulate mice enables investigation of the genomic factors that regulate outflow physiology [15–20]. The considerable disadvantage of mouse eyes is their diminutive size, being approximately 3% of the volume of AH compared to human eyes [21, 22], which corresponds to extremely low flow rates, on the order of 50 *nl*/*min* at physiological IOP. This makes the assessment of outflow facility extremely sensitive to uncertainties in the methods of measurement and data analysis.

The present study describes *iPerfusion*^™^, a new paradigm for the measurement of outflow facility by ocular perfusion, incorporating measurement techniques, data analysis and presentation methods. Although the present study focuses on enucleated mouse eyes, the system can be scaled up for facility measurements in any species, and with minor modifications may be applied *in vivo*. To provide context, a brief review of existing techniques is provided in the following section.

### Existing techniques to measure outflow facility

The commonly used approach for measuring outflow facility is based on mass conservation of the flow entering and exiting the eye during an *in vivo* perfusion according to:

$$Q_{in}+Q=C\;\left(P-P_{e}\right)+Q_{0}$$ (1)

which is known as the modified Goldmann equation [23]. *Q*_*in*_ is the rate of AH secretion, *Q* is the flow rate into the eye from the perfusion apparatus and *Q*_0_ is the pressure-independent outflow. *P* is the intraocular pressure and *P*_*e*_ is the pressure in the episcleral vessels (into which the AH drains). In this form, *C* is the total outflow facility, comprising both conventional outflow and any pressure-dependent components of unconventional outflow and AH secretion (pseudofacility) [23]. Herein we will use the term ‘facility’ to indicate *C*, for simplicity. In order to calculate facility, *Q*_0_ and *Q*_*in*_, *P*_*e*_ and *C* itself are often assumed to be pressure independent (thereby tacitly assuming a linear *Q* − *P* relationship). Under these assumptions, two measurements of *P* and *Q* are thus sufficient to estimate the facility according to the two-step perfusion protocol [24]:

$$C_{\text{lin}}=\frac{Q_{\text{II}}-Q_{\text{I}}}{P_{\text{II}}-P_{\text{I}}}$$ (2)

where the subscripts I and II denote measurements at two different pressures, and *C*_lin_ is a pressure independent facility, based on the assumption of a linear *Q* − *P* response. Alternatively, for the case of enucleated eyes, *Q*_*in*_ and *P*_*e*_ are zero, hence Eq 1 reduces to

$$Q=C_{\text{lin}}P+Q_{0}$$ (3)

In order to provide a more robust method than using Eq 2, it has become common in mouse eye perfusions to measure multiple (3–5) points and fit a straight line to the *Q* − *P* relationship, assuming that the facility, given by the slope of the line, *C*_lin_, is pressure independent. The unconventional outflow is sometimes estimated from the intercept using this approach [11–13, 20, 21, 25], although others note that the validity of the method is questionable [26–28]. Fitting a straight line to the data to estimate facility is only appropriate if *C* is independent of pressure and *Q*_0_ has a finite value. The validity of these assumptions in the context of enucleated mouse eyes will be investigated in the present study.

A number of methodologies for assessing the flow rate into mouse eyes from perfusion systems have been reported in the literature. These may be grouped into (1) syringe pump based systems, in which the flow into the eye is prescribed and the pressure in the eye measured, (2) pressure-decay based systems, in which the flow rate and pressure are inferred based on the change in height of a small diameter fluid reservoir, and (3) constant pressure systems, in which a large diameter fluid reservoir provides a relatively constant eye pressure and the flow rate is measured, for example, based on the pressure drop along a capillary tube of known resistance.

#### Syringe pump based systems

In these systems, a syringe pump is used to deliver a nominal flow rate, and the pressure in the eye is monitored. Syringe pumps function via a lead screw driven by stepper motor, and at the low flow rates required for mouse eye perfusions, the finite steps may introduce pulsations in the flow. These pulsations are proportional to the square of the inner diameter of the syringe and the step size, and thus use of appropriately small syringe sizes and high resolution pumps is critical. The simplest pump based approach is to apply a constant flow rate and allow the system to naturally reach a steady state pressure for each flow rate [12], although this may take a very long time (30–40 min) [16]. In order to decrease the time to reach a stable condition, active control of the flow rate can be used to regulate the measured pressure, often with a pre-pressurisation stage at each step to further improve the transient response of the system [11, 16, 21, 25, 29, 30].

In pump based systems, a number of flow or pressure steps are analysed, and a straight line is fitted to the steady state values. The selection of steady state is often not clearly defined and is prone to subjectivity, particularly in active control systems. Furthermore, very few studies using pump based systems to measure outflow facility validate the pump output or elucidate the control algorithm used to obtain and regulate the desired pressure (in systems with active control), which complicates verification of the results by other research groups.

#### Pressure-decay based systems

In pressure-decay based systems, a small diameter (*D*) reservoir open to atmospheric pressure is used to apply a hydrostatic pressure to the eye. As fluid flows from the reservoir into the eye, the reservoir height decreases over time, *t*. In the classic ‘two-step’ perfusion method developed for primates [24], the flow rate is measured based upon the change of mass, *m*, of the fluid reservoir, but this would not be suitable for mouse eyes due to the low flow rates (over a 10 minute period under physiological conditions, the change in mass for an average mouse eye would be less than 1 *mg*). Aihara et al. [21], estimated the flow rate by observing the change in height of the fluid reservoir, *h*_*r*_, and related this value to the volume perfused into the eye over a certain time period. Camras et al. [31] instead related the pressure gradient (based on a straight line fit to *P* vs *t*) to the change in height, so as to estimate a flow rate. These three approaches are mathematically equivalent and can be written as:

$$Q=\frac{1}{\rho }\frac{dm}{dt}=\frac{\pi D^{2}}{4}\frac{dh}{dt}=\frac{\pi D^{2}}{4\rho g}\frac{dP}{dt}$$ (4)

where *ρ* is the density of the fluid in the reservoir and *g* is the gravitational acceleration. In all three forms of Eq 4 (based on change in weight, height or pressure), the flow rate is inferred based on the derivative of a measured parameter, generally estimated from two time points. Consequently, instantaneous measurements of the flow rate are not possible, and the methods yield only an average value over the evaluated time period, typically on the order of minutes. Furthermore, as the reservoir height is used to infer both the flow rate and the pressure, a trade-off exists wherein a higher resolution in measurement of the flow rate, achieved by reducing *D* or increasing the time period, is accompanied by a greater change in the applied pressure. Additional sources of error in these methods include estimation of the height of the reservoir, non-linearity in the pressure sensor or weight measurement, inaccuracies in specification of *D* (as the flow rate scales with *D*^2^) and evaporation. For these reasons, along with the uncertainty in differentiating inherently noisy experimental data [32], it is preferential to acquire data on a parameter that is proportional to the flow rate itself [33].

#### Constant pressure systems

In constant pressure systems, a reservoir with a large diameter can be used to apply a relatively constant pressure, whilst measuring the flow rate directly. Typically, the flow rate is calculated based on the pressure drop along a capillary tube of known diameter, located upstream of the eye, using the Hagen-Poiseuille law. Ethier et al. [33] used a differential pressure sensor to measure the pressure drop across a 150 *μm* capillary tube, with a second pressure sensor to measure the IOP. The pressure drop across a capillary tube is highly dependent on temperature (water viscosity decreases by more than 30% as temperature increases from 20 to 37°*C*, ≈2% per degree), hence in their system Ethier et al. [33] immersed the capillary tubing in a temperature controlled water bath. The system was tested in the range of facilities relevant to human eyes (≈200 *nl*/*min*/*mmHg*). Kee et al. [34] proposed a modified version of this system in which a number of components added for accuracy were omitted in the interest of simplicity. They tested their system using rat eyes (*C* ≈ 50 *nl*/*min*/*mmHg*). This approach has not been adopted for studies in mouse eyes, which have considerably lower facilities (*C* ≈ 5 *nl*/*min*/*mmHg*). In addition to the requirement for temperature regulation and accurate pressure measurement, a small diameter capillary would be needed to generate an accurately measurable pressure drop (≈75 *μm* or less). From a practical perspective, such small diameter capillaries are prone to blockages and biofilm formation, which would drastically alter the hydrodynamic resistance of the tube and hence the estimation of the flow rate.

In the present study, we use a large diameter reservoir and commercially available thermal flow sensor, which enables direct, repeatable, and accurate measurement of the flow rate. The flow rate is calculated based on thermal gradients along a capillary tube, induced by a small heating element. Knowledge of the applied pressure, the resistance of the flow sensor and the flow rate through the sensor provides redundant information, which can be used to monitor the functioning of the flow sensor itself (for example, to detect blockages).

In summary, measurements of outflow facility in mice are technically challenging and extremely sensitive to small errors. Although a number of techniques have been described in the literature, none have been sufficiently validated and all have considerable limitations. To overcome these challenges, we have developed the *iPerfusion* system.

## Methods

*iPerfusion* comprises hardware, control software, protocol, data analysis and statistical methodologies, and graphical representations, that together maximise the scientific value of each eye perfused. This section is subdivided into data acquisition (including validation of the system) and statistical analysis.

### Data acquisition

#### Experimental setup

**Fig 1:**
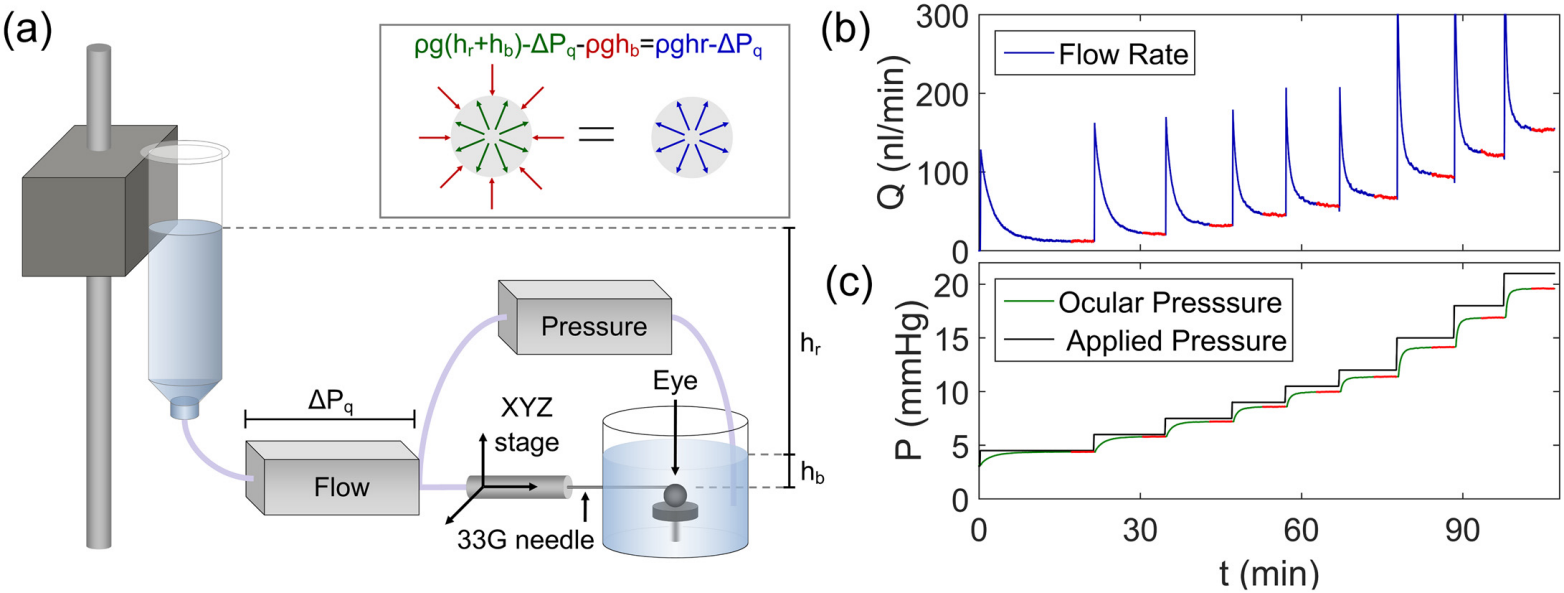
The *iPerfusion* system. (a) Schematic of the experimental setup. Inset shows internal (green), external (red) and resultant (blue) pressures acting on the eye. Flow (b) and pressure (c) traces from a sample mouse eye perfusion. Red highlighted regions show steady-state periods, over which data were averaged.

The hardware consists of three main components; an actuated reservoir, a thermal flow sensor and a differential pressure transducer (Fig 1a). On either side of the pressure transducer, a manifold (not shown) is used to control the flow path, in order to switch between configurations required for sensor calibration and system validation. The pressure applied to the system is controlled using a 10 *ml* reservoir, filled with water. The reservoir is coupled to a vertically mounted linear actuator (L35; Nanotec, Germany). The fluid passes from the actuated reservoir through the flow sensor (SLG64-0075; Sensirion AG, Switzerland), which comprises a 75 *μm* diameter glass capillary, onto which two temperature sensing elements are bonded on either side of a heating element. Based on knowledge of the flow characteristics (Poiseuille) and the heat transfer properties of water, the flow rate can be calculated in the range −5000 to 5000 *nl*/*min*. The pressure difference across the outflow pathway is measured using a silicon micromachined wet-wet differential pressure transducer (0–50 *mmHg*; PX409; Omegadyne, USA). Supporting Information 1 (S1 File) provides analysis of the accuracy of the flow and pressure sensors. The results show that the uncertainties, in the range appropriate for mouse eye perfusions, are given by ±4 *nl*/*min* and ±0.024 *mmHg* (two standard deviations (SD)) for the flow and pressure sensors respectively.

The eye is completely submerged in a relatively large volume of phosphate buffered saline (PBS; 20–40 *ml*), which is regulated at 35 ± 0.5°*C*. Submersion ensures that the temperature and hydration of the eye remain constant throughout the experiment. The submersion depth has no effect on the pressure drop across the outflow pathway for the following reason. As shown in Fig 1a, hydrostatic pressure acting on the outside of the eye is given by *ρgh*_*b*_, where *h*_*b*_ is the submersion depth of the eye. For the case of zero flow into the eye, the pressure inside the eye is given by *ρg*(*h*_*b*_ + *h*_*r*_), where *h*_*r*_ is the height of the reservoir relative to the surface of the fluid in the eye bath. The difference between the internal and external pressures, is given by *ρgh*_*r*_, which we refer to as the applied pressure, *P*_*a*_. As the flow sensor consists of a small diameter capillary, it has a hydrodynamic resistance that results in a pressure drop, Δ*P*_*q*_ = *Q*(*t*)/*C*_*q*_ across the sensor, in the general case of non-zero flow. The measured pressure in the eye, *P*(*t*) is therefore related to the applied pressure, *P*_*a*_, according to

$$P\;\left(t\right)=\rho g\;\left(h_{r}+h_{b}\right)-\Delta P_{q}\;\left(t\right)-\rho gh_{b}=P_{a}-Q\;\left(t\right)/C_{q}$$ (5)

which is independent of submersion depth. Due to its location downstream of the flow sensor, the wet-wet differential pressure transducer directly measures *P*(*t*), and therefore would not be affected by changes in *h*_*b*_, for example due to evaporation from the heated eye bath or reservoir.

For the present study, two duplicates of the experimental setup were used, so that both eyes from a given mouse could be measured simultaneously, eliminating the influence of post-mortem time between paired eyes. The time between enucleation and cannulation was generally less than 30 minutes.

#### Experimental protocol

The basic protocol for each perfusion comprises three sections: calibration/testing of the system, cannulation of the eye and acclimatisation, and a multi-step perfusion regimen.

For each day of experiments, the pressure sensor was calibrated using an automated 8-point calibration with the automated linear actuator, which has a resolution of 1.25 *μm* per step. Subsequently, the resistance of the flow sensor was measured to ensure that it was blockage free. Prior to each perfusion, the resistance of a glass capillary of known resistance (comparable to a mouse eye) was measured to confirm that the system was functioning properly. The perfusion tubing (downstream of the flow sensor) and needle were then filled with perfusate and the resistance of the needle was measured before cannulating the eye. Note that, as the fluid in the system was water, there was a water-perfusate interface in the perfusion tubing. However, given that the volume of the tubing (≈2*ml*) was large compared to the total volume perfused in an experiment (≈25*μl*), neither advection nor diffusion could have altered the perfusate entering the eye. If any of the readings from the tests were unexpected, for example if the system time-response was too long or measured resistances were not correct, leaks/bubbles/blockages were identified and removed before the ocular perfusion commenced.

All animal experiments were done *ex vivo* in accordance with the Animals (Scientific Procedure) Act with the authority of a UK Home Office project licence (PPL 70/7306). All perfusions were carried out on 10–14 week old male C57BL/6J (B6) mice (Charles River UK, Ltd.), that were euthanised via cervical dislocation. All mice were fed *ad libitum* and housed in clear cages at 21°*C* with a 12 hour light-dark cycle (lights on at 7AM).

After enucleation, the eyes were stored in PBS at room temperature to await perfusion. Each eye was then affixed to a platform inside the heated bath with a small amount of cyanoacrylate glue (Loctite, UK). An XYZ micromanipulator (World Precision Instruments, USA) was used to cannulate the eye via the anterior chamber with a 33 gauge needle (Nanofil, World Precision Instruments, USA) under a stereomicroscope. The control perfusate was DBG: Dulbecco’s PBS containing divalent cations, supplemented with 5.5 *mM* glucose and passed through a 0.2 *μm* filter. After cannulation, the bath was filled with PBS to fully immerse the eye and the temperature was raised to 35°*C*. The applied pressure was held at 9 *mmHg* for a period of 30–45 minutes to allow the eye to acclimatise to the pressure and temperature environment.

After acclimatisation, a nine-step perfusion protocol was carried out, consisting of applied pressures of 4.5, 6, 7.5, 9, 10.5, 12, 15, 18 and 21 *mmHg*. A sample perfusion tracing is shown in Fig 1b and 1c.

In order to avoid a subjective element in the definition of steady state at each pressure step, a steady state condition was defined and automatically monitored by the perfusion software. A parameter *Γ*(*t*) = *Q*(*t*)/*P*(*t*) was continuously evaluated and *dΓ*/*dt* was estimated by linear regression over a moving window of 5 minutes. When |*dΓ*/*dt*| was continuously less than 0.1 *nl*/*min*/*mmHg*/*min* for one minute, the system was considered to be at steady state. The measured flow and pressure were then averaged over the four previous minutes to yield *Q*_*j*_ and *P*_*j*_ respectively, for pressure step *j*. The red lines in Fig 1b and 1c indicate these averaging periods. This approach ensures that the measured facility changes by less than 0.4 *nl*/*min*/*mmHg* over the averaging period. For the eyes in the present study, the median change in facility over the averaging window was 0.16 *nl*/*min*/*mmHg*.

#### System validation

In order to ascertain the accuracy of the pressure and flow sensors, independent tests were carried out and are described in S1 File. A series of *in vitro* tests were then carried out to investigate the accuracy of the system in measuring facility (hydrodynamic conductance). A number of lengths of thick walled borosilicate glass capillaries (CM Scientific, UK) with inner diameter of 51 ± 5 *μm* were used to create resistances comparable to the outflow resistances measured in mouse eyes. The capillaries were cut to lengths, *L*_*c*_, of approximately 50, 100 and 150 *mm*. Additional lengths of 250 *mm* and 350 *mm* were achieved by adding capillaries in series. For each length of capillary, a 7 step perfusion (steps of 4 *mmHg* starting at 4 *mmHg*) was carried out three times. For each pressure step *j*, the uncertainty in the pressure was negligibly small (see S1 File), and the uncertainty in the flow rate (given as a variance), $s_{Q,j}^{2}$, was calculated by adding the variance of the measured flow rate over a 30 second window, $s_{Q\text{ave}}^{2}$, and the uncertainty of the flow sensor, $s_{Q\text{sens}}^{2}$. The value of the hydrodynamic conductance of each length of capillary, *C*, was calculated for each test by fitting the line *Q* = *CP* to the *Q*_*j*_ and *P*_*j*_ values, using weighted regression with weights defined according to $1/s_{Q,j}^{2}$.

Data based on regression analysis are presented as the mean ± the margin of error at a 95% confidence level, (*ME*_95_), which is defined as the half-width of the 95% confidence interval (CI). When analysing the variability within a sample, two standard deviations will be reported, which indicate the range of values in which 95% of the population are expected to lie: we will refer to this as ‘two-sigma’ herein.

**Fig 2:**
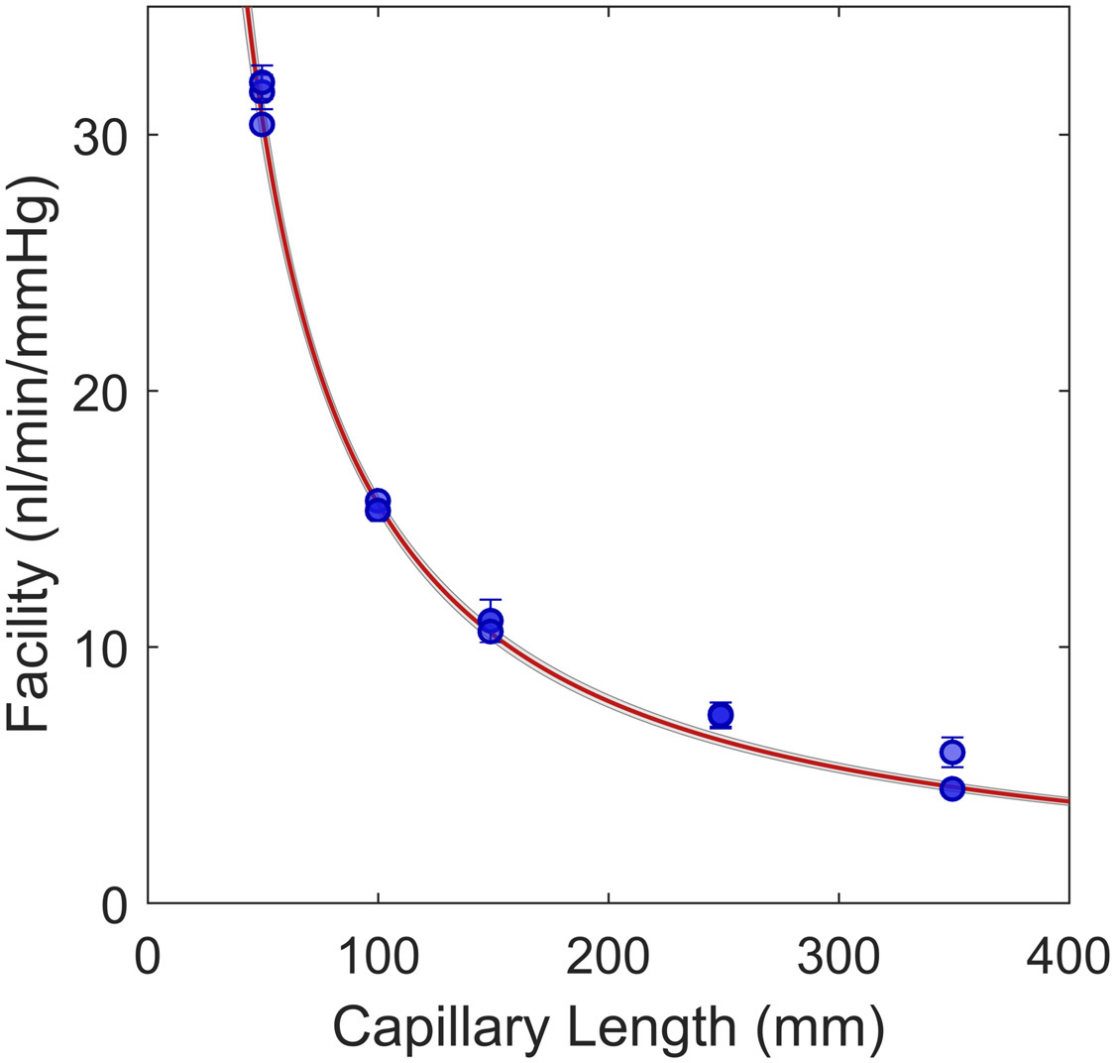
*In vitro* validation of the system. Data points show the ‘facility’ (hydraulic conductance) of various lengths of glass capillary with 95% confidence intervals. The fit in red is a linear relationship between hydraulic resistance and length, with 95% confidence bounds shown in grey. Deviations from the fit can be used to estimate the accuracy of facility measurement using *iPerfusion*.

Fig 2 shows the measured *C* with the 95% CI for each of the repeated experiments at each capillary length. The red line and thin grey lines show the best fit and 95% confidence bounds, respectively, to the equation *C* = 1/(*R*_*L*_ *L*_*c*_+*R*_0_), where *R*_*L*_ is the resistance per unit length and *R*_0_ represents the resistance of the system downstream of the flow sensor. This model is used as hydrodynamic resistance (the reciprocal of conductance) is proportional to length. The model was fitted using weighted regression, with weights defined according to $1/s_{C}^{2}$, where *s*_*C*_ is assumed to be equivalent to the 68% ME on *C*. The resistance per unit length was found to be *R*_*L*_ = 0.627 ± 0.029 *mmHg*/*μl*/*min*/*mm* (mean ± *ME*_95_), while *R*_0_ = 1.50 ± 2.25 *mmHg*/*μl*/*min* was not significantly different from zero. To confirm that the system was measuring correct values in absolute terms, the diameter of the capillary based on Poiseuille flow (at 22°*C*) was calculated from *R*_*L*_ and its confidence intervals. The predicted diameter was 52.8 ± 0.6*μm*, which is within the manufacturer’s specification (see above). The root mean square deviation from the best fit was 0.8 *nl*/*min*/*mmHg*, and thus a conservative estimate of two-sigma for the measurement of facility could be given as ±1.6 *nl*/*min*/*mmHg* over the range of facilities relevant for mouse eyes.

These *in vitro* capillary tests provide a frame of reference to establish that facility measurements can be made accurately and reproducibly within the range appropriate for mouse eyes using *iPerfusion*. It must be stressed, however, that the accuracy when measuring outflow facility in real eyes will not necessarily be the same as that measured with *in vitro* capillaries. While some sources of uncertainty overlap, such as the flow rate measurement, the measurement of biological tissues will inevitably involve additional, and likely greater, uncertainties, such as those associated with tissue variability and with interfacing mechanical and biological systems.

### Statistical analysis

In ocular perfusion studies, statistical analysis is generally carried out using hypothesis testing, where the statistical significance of an experimental treatment is assessed using, for example, Student’s two sample *t*-test (paired or unpaired). However, due to biological variability and the limitations of any experimental measurement, each data point analysed will have some degree of uncertainty associated with it. The standard *t*-test, implicitly assumes that the measurement uncertainty is equal for all samples. The validity of the statistical analysis can be improved by accounting for uncertainty in the acquired data. To this end, we have developed a statistical approach, including the ‘weighted *t*-test’, a modification of Student’s *t*-test that includes variable uncertainties between data points.

Here we present an overview of the statistical analysis; a complete description of the mathematics is given in S2 File. Although this approach has been developed for processing eye perfusion data, the weighted *t*-test is applicable to any statistical analysis in which a *t*-test might normally be used, but in which individual measurement uncertainties differ between cases. The analysis method additionally provides useful estimates of the sample variability and treatment effect.

A nomenclature listing all mathematical terms used in the analysis is provided in S3 File.

#### Statistical distributions

Any parametric statistical analysis requires an assumption of the underlying probability distribution. In order to use the *t*-test and many of the tools utilised in the following analysis, the data should be sampled from a normal distribution. However, as reviewed by Limpert et al. [35, 36], for many real world applications, a normal distribution is often not appropriate. We posit that this is also the case for facility, for the following reasons.

For the outflow facility of mouse eyes reported in the literature, the standard deviation is often of similar magnitude to the mean [17, 20, 21, 31], such that range of facilities for a sample of mice (the lower limit of which can be estimated as the mean minus two standard deviations), would predict some very low, unphysiological, facilities according to the normal distribution. Furthermore, facility is inherently multiplicative, rather than additive (for example, doubling the resistance for a given flow rate would double the pressure drop across the outflow pathway). From these points, we conclude that outflow facility should be treated as a lognormally distributed variable, as will be confirmed in the Results section. Therefore, by analysing the logarithm of *C*, *Y* = ln(*C*), we may use statistical tools based on the normal distribution.

Unlike facility, the measured flow rate and pressure data are normally distributed variables, as uncertainties in these measurements occur due to electrical and external noise. The calculation of facility will be carried out by fitting a model to the measured flow rate and pressure data using weighted regression analysis, which requires normally distributed data. However, where the output of the regression analysis yields a lognormally distributed variable, such as facility, further consideration is required.

Consider fitting Eq 3 to the measured pressure and flow data, for a given eye, for which the best estimate of *C*_lin_ ± *s*_*C*_lin__ would be obtained. If the variable *C*_lin_ was normally distributed, it could be described according to *C*_lin_ ± *s*_*C*_lin__. However, as discussed, it is not *C*_lin_, but *Y*_lin_ = ln(*C*_lin_) that is normally distributed, and we therefore require *Y*_lin_ ± *s*_*Y*_lin__. As ln(*C*_lin_ − *s*_*C*_lin__) and ln(*C*_lin_+*s*_*C*_lin__) are not equidistant from ln(*C*_lin_), it is not possible to state ±*s*_*Y*_lin__ as a function of *s*_*C*_lin__. Rather than fitting Eq 3, it is possible to directly calculate *s*_*Y*_lin__ from the regression, by fitting the model

$$Q=e^{Y_{\text{lin}}}\;P+Q_{0}$$ (6)

which yields *Y*_lin_ ± *s*_*Y*_lin__, where the value of *C*_lin_ is equal to e^Y_lin_^.

With regards to the *Q*_0_ term, it is worth noting that as *Q*_0_ can take on negative values, it cannot be lognormally distributed. It is therefore reasonable to assume that *Q*_0_ is normally distributed, and the values of *Q*_0_ ± *s*_*Q*_0__ will be appropriate for further analysis.

#### Sources of uncertainty

There are five sources of uncertainty that contribute to variability in ocular perfusion measurements. We use the term *s*^2^ to indicate an uncertainty in the form of a sample variance. Variances are used as they may be added to combine sources of uncertainty.

1. Measurement of pressure and flow rate: due to non-linearity, irrepeatability and hysteresis present in all transducers, each measurement has an associated degree of uncertainty that should be accounted for in the analysis, $s_{\text{sens}}^{2}$ (sensor uncertainty). Additionally, there is a component of uncertainty due to averaging the signal over a period of time, $s_{\text{ave}}^{2}$ (averaging uncertainty). For the flow measurement (subscript *Q*), the total uncertainty for each pressure step *j*, can be calculated according to
  $$s_{Q,j}^{2}=s_{Q\text{sens}}^{2}+s_{Q\text{ave},j}^{2}$$
  For the present system, we found that for the pressure measurements (subscript *P*) the total uncertainty $s_{P,j}^{2}$, is negligibly small (see S1 File). Thus from the raw data we obtain the steady state values *P*_*j*_, *Q*_*j*_ and the uncertainty in the flow rate, $s_{Q,j}^{2}$.
2. Fitting a model to the data: the facility is calculated by fitting a model to the flow rate and pressure data using weighted least-squares regression between *Q*_*j*_ and *P*_*j*_, with the weights defined as $1/s_{Q_{j}}^{2}$. For each eye, *i*, each of the regression parameters in the model has an associated variance based on its confidence interval, $s_{\text{reg},i}^{2}$ (regression uncertainty).
3. Intra-individual variability: when using a paired experimental design, it is generally implicitly assumed that in the absence of a treatment, no difference would be observed. However, in complex measurements, a certain amount of deviation from the ideal case is to be expected. To quantify this, we introduce the intra-individual variability between contralateral eyes, $s_{\text{con}}^{2}$. This parameter inherently includes both the intrinsic biological variability in outflow facility between contralateral eyes and the added uncertainty associated with interfacing the eye with the perfusion system (cannulation), as numerically these are inseparable. For a given experimental system and sample population, the value of $s_{\text{con}}^{2}$, can be estimated by carrying out paired perfusions on untreated contralateral eyes, as described in detail in the Results section and in S2 File. In order to do this, it is assumed that the total variability in the difference in facility between the paired eyes, $s_{\text{dif}}^{2}$, is made up of contributions from the average uncertainty in the regression, $\bar{s_{\text{reg}}^{2}}$, and $s_{\text{con}}^{2}$. Therefore, the intra-individual variability is given by
  $$s_{\text{con}}^{2}=s_{\text{dif}}^{2}-2\bar{s_{\text{reg}}^{2}}$$
  where the factor 2 arises due to regression uncertainty from both eyes of each pair. Although $s_{\text{con}}^{2}$ does not numerically affect the outcome of the weighted *t*-test, knowledge of its value is necessary for estimating the variability of a given treatment in paired analyses, assists in the visualisation of uncertainties for pairs of eyes, and is beneficial for understanding the limitations of a perfusion system.
4. Inter-individual variability: when using an unpaired experimental design there will be a variability in outflow facility between individuals for a given population, $s_{\text{pop}}^{2}$, termed the inter-individual variability. The value of $s_{\text{pop}}^{2}$ can be estimated by calculating the total variance of measured facilities over a given sample, $s_{\text{tot}}^{2}$, and assuming that it comprises both $s_{\text{pop}}^{2}$ and $\bar{s_{\text{reg}}^{2}}$. The inter-individual variability is then given by
  $$s_{\text{pop}}^{2}=s_{\text{tot}}^{2}-\bar{s_{\text{reg}}^{2}}$$
  As with the $s_{\text{con}}^{2}$ term, this inherently includes an uncertainty associated with interfacing the eye with the perfusion system.
5. Treatment variability: in experiments where the effect of a treatment is evaluated using either paired or unpaired analysis, there will be a variability in the efficacy of the treatment, $s_{\text{tre}}^{2}$. This parameter can be estimated as the difference in the variability between the treated and control samples. Large values of $s_{\text{tre}}^{2}$ imply that the treatment effect varies considerably between individuals, for example if there are subpopulations of ‘responders’ and ‘non-responders’. Calculation of very small or negative values of $s_{\text{tre}}^{2}$ would indicate that the additional uncertainty due to the treatment is negligible compared to the inherent variability present in untreated eyes. Equations for calculating $s_{\text{tre}}^{2}$ are provided in the next section, and the derivations are presented in S2 File.

#### Central tendency and measures of spread using weighting

Typically, the arithmetic mean, standard deviation and standard error on the mean (SEM) are used as measures of the central tendency and spread of the data. However, these values do not incorporate the variable uncertainty for each measurement. In order to account for measurement uncertainty and thus provide improved estimates of the sample statistics, the weighted arithmetic mean (WAM) is used. To calculate the WAM, each value is weighted relative to the reciprocal variance of the total uncertainty for each pair or individual eye (see below for details). Thus data points with large uncertainty contribute less to the weighted average, making the calculation more robust. The weighted standard error on the mean (WSEM) can be calculated as the square root of the variance on the WAM, and the square root of the unbiased weighted sample variance gives the weighted standard deviation (WSD). A brief description is given in the following section, refer to S2 File for details and derivation.

Paired data: for paired data, we first calculate the difference in the parameter of interest for each pair, and then calculate the *average of the differences*. For facility, we must use the log transform *Y* = ln(*C*). The difference for a pair of eyes *p* is then given by *Z*_*p*_ = *Y*_2,*p*_ − *Y*_1,*p*_, where the subscripts 1 and 2 refer to the control and treated eyes respectively. The weight for each *Z*_*p*_ is given by the reciprocal of the total uncertainty for each pair

$$s_{Z_{p}}^{2}=s_{\text{tot},Z}^{2}+s_{\text{reg},p,1}^{2}+s_{\text{reg},p,2}^{2}-2\bar{s_{\text{reg}}^{2}}$$

where $s_{\text{tot},Z}^{2}$ is the unweighted variance of *Z*_*p*_. The WAM, $\bar{Z}$, can be calculated as the weighted arithmetic mean of *Z*_*p*_. The variance of $\bar{Z}$ is $s_{\bar{Z}}^{2}$ and the unbiased weighted sample variance is $s_{Z}^{2}$. The treatment variability $s_{\text{tre}}^{2}$ is estimated using

$$s_{\text{tre}}^{2}=s_{Z}^{2}-s_{\text{con}}^{2}-2\bar{s_{\text{reg}}^{2}}$$

Unpaired data: for unpaired data, we calculate the averages for each population *A* and *B*, and then calculate the *difference between the averages*. For each eye, *i*, the weight is given by the reciprocal of the total uncertainty for each eye

$$s_{Y_{i}}^{2}=s_{\text{tot}}^{2}+s_{\text{reg},i}^{2}-\bar{s_{\text{reg}}^{2}}$$

where $s_{\text{tot}}^{2}$ is the unweighted variance of *Y*_*i*_. $\bar{Y}$, $s_{\bar{Y}}^{2}$ and $s_{Y}^{2}$ can then be calculated for each population sample. $\bar{Z}$ is then calculated as the difference between the WAM of the two population samples, $\bar{Z}={\bar{Y}}_{B}-{\bar{Y}}_{A}$. The variance of $\bar{Z}$ can be calculated by adding the variances from the two population sample, $s_{\bar{Z}}^{2}=s_{{\bar{Y}}_{A}}^{2}+s_{{\bar{Y}}_{B}}^{2}$. The treatment variability $s_{\text{tre}}^{2}$ is estimated by assuming $s_{\text{pop}}^{2}$ is equal for both samples, and thus where the treated population sample has a larger variance, it can be attributed to the treatment, hence:

$$s_{tre}^{2}=s_{Y_{B}}^{2}-s_{Y_{A}}^{2}-\left(\bar{s_{\text{reg},B}^{2}}-\bar{s_{\text{reg},A}^{2}}\right)$$

using the values of $\bar{s_{\text{reg}}^{2}}$ for each population.

Lognormal variables: for a given population, the variable $\bar{Y}$ corresponds to the average logarithm of the outflow facility (expressed in dimensionless units). As it is preferable to report results in units of facility, we must take the exponential of $\bar{Y}$, yielding the geometric mean, ${\bar{C}}^{*}=e^{\bar{Y}}$, with $s_{\bar{C}}^{*}=e^{s_{\bar{Y}}}$. Likewise, $s_{C}^{*}=e^{s_{Y}}$. Note that the * denotes the use of geometric variables. When moving from the additive (log) domain, in which we describe $\bar{Y}\pm s_{\bar{Y}}$, into the multiplicative (linear) domain, the ‘plus or minus’ (±) term no longer applies, and is replaced by ‘times or divide by’ (^×^/) [36]. Therefore, $\bar{Y}\pm s_{\bar{Y}}$ is expressed as ${\bar{C}}^{*}\;{}^{\times }/\;s_{\bar{C}}^{*}$. Note that when using $\pm 2s_{\bar{Y}}$, the lognormal equivalent becomes $\;{}^{\times }/\;s_{\bar{C}}^{*2}$, as $e^{2s_{\bar{Y}}}={\left(e^{s_{\bar{Y}}}\right)}^{2}=s_{\bar{C}}^{*2}$.

For both paired and unpaired data, the variable $\bar{Z}$ represents the average difference in the logarithm of the facility, *Y*. A more intuitive variable is the average fold change given by ${\bar{D}}^{*}=e^{\bar{Z}}$. This can be described easily for unpaired data wherein, $\bar{Z}={\bar{Y}}_{B}-{\bar{Y}}_{A}$. Converting into the multiplicative domain yields ${\bar{D}}^{*}=e^{\bar{Z}}={\bar{C}}_{B}^{*}/{\bar{C}}_{A}^{*}$, which is the proportional change in the average facility. Therefore $s_{\bar{D}}^{*}=e^{s_{\bar{Z}}}$ describes the spread in the estimate of ${\bar{D}}^{*}$, and hence how well the average fold change is known.

#### Reporting statistical results

For each reported statistic, we provide a weighted mean (geometric or arithmetic for lognormal or normal variables respectively), along with the 95% confidence interval on the weighted mean. In addition we provide an indication of the spread or variability in the population. For discussion of why we report the statistics in this way, please see S2 File, Section S2–6.

Statistical results for a lognormally distributed variable, such as facility, will be reported as

$${\bar{C}}^{*}\;{}^{\times }/\;ME_{{\bar{C}}^{*},95}\left(s_{\text{pop}}^{*2}\right)$$

where $ME_{{\bar{C}}^{*},95}$ is the margin of error given by the half-width of the 95% confidence interval on the estimate of ${\bar{C}}^{*}$. The confidence interval on ${\bar{C}}^{*}$ can be calculated as $\left[{\bar{C}}^{*}/ME_{{\bar{C}}^{*},95},{\bar{C}}^{*}\times ME_{{\bar{C}}^{*},95}\right]$. The term $s_{\text{pop}}^{*2}$ corresponds to two-sigma, an estimate of the range that would encompass 95% of the facility values within the population ($s_{\text{pop}}^{*2}=e^{2s_{\text{pop}}}$). Note that the margin of error and two-sigma values are multiplicative.

Statistical results for a normally distributed variable, such as *Z*, will be reported as

$$\bar{Z}\pm ME_{\bar{Z},95}\;\left(2s_{\text{tre}}\right)$$

where 2*s*_tre_ is two-sigma, gives an estimate of the range that encompasses 95% of the treatment variability. The proportional change will be reported as

$${\bar{D}}^{*}\;{}^{\times }/\;ME_{{\bar{D}}^{*},95}\;\left(s_{\text{tre}}^{*2}\right)$$

In addition to ${\bar{D}}^{*}$, it may be preferable to report the average percentage change, given by $({\bar{D}}^{*}-1)\times 100\%$. The confidence bounds for the percentage change are given by $({\bar{D}}^{*}\;{}^{\times }/\;ME_{{\bar{D}}^{*},95}-1)\times 100\%$ and thus it is not possible to state them in terms of a simple ^×^/. This is also the case for the treatment effect. Thus, when reporting a percentage change, we will provide the confidence interval, and/or the range bounded by two-sigma directly. For example, a proportional change of ${\bar{D}}^{*}=1.5$ with $ME_{{\bar{D}}^{*},95}=1.1$ and $s_{\text{tre}}^{*2}=1.25$ could be reported as a 50% increase, with confidence interval [36,65%] and a treatment effect between 20% and 88%.

#### Hypothesis testing

Student’s *t*-test yields a *p*-value, which is an estimate of the probability that the null hypothesis ($\bar{Z}=0$) is true, based on a *t*-statistic and an estimate of the number of degrees of freedom, *ν*. The ‘weighted *t*-test’ described in S2 File is similar but incorporates uncertainties by calculating the *t*-statistic as the ratio of the weighted mean to the square root of its weighted variance, $t=\bar{Z}/s_{\bar{Z}}$. Due to the weighting of each data point, *ν* will be less than that normally used in a standard *t*-test (for example *ψ* − 1 for a paired *t*-test with *ψ* pairs). For the weighted statistics used in this study, *ν* can be approximated using the Welch-Satterthwaite equation (see S2 File for details).

#### Selection criteria

Three stages of selection criteria must be passed in order for a given perfusion to be included in further analysis. These approaches were designed to be as robust and as objective as possible.

1. A benefit of the *iPerfusion* system is that it enables continuous monitoring of the pressure and flow rate throughout the perfusion. Therefore, complications due to a poor cannulation, such as the cornea blocking the needle or poor sealing around the needle tip, become readily apparent. Cases in which the tracings deviate significantly from the prototypical tracing shown in Fig 1b and 1c, were thus excluded from further analysis. This process was carried out prior to calculating the facility, in order to minimise bias.
2. In response to a step increase in applied pressure, the measured pressure typically displays an exponential decay-type response, settling to a constant pressure over several minutes. In some eyes, typically at higher pressures, the applied pressure did not reach an apparent steady state, but slowly decreased over a longer time. To detect and eliminate these cases, the time constant of the pressure decay was estimated by fitting a single exponential, and any pressure trace that varied by more than 0.2 *mmHg* after 6 time constants (at which time the system should be within 0.3% of its steady state value), was considered not to have a definable steady state condition. In such cases, the non-steady state pressure step and all subsequent pressure steps were eliminated from the regression analysis. Cases with fewer than four stable pressure steps were omitted on the basis of being insufficient for regression analysis using a model with two free parameters. The median number of pressure steps used per eye in the present study was 8.
3. For paired data, putative outliers in the observed *Z* values were identified. A non-parametric approach was used, whereby the median med(*Z*) was calculated as a robust estimate of the central value. The median absolute deviation (MAD)
  $$\text{MAD}=1.4826\;\text{med}\left|Z_{p}-\text{med}\left(Z\right)\right|$$ (7)
  was used as a robust estimator of the sample standard deviation (the 1.4826 is dependent on the assumption that the underlying distribution is normal). Outliers were classified as data points for which the measured value including the uncertainty exceeded 2.5 MAD from the median [37]. Thus, data points greater than the median were omitted from the analysis if *Z*_*p*_ minus the regression uncertainty exceeded the median plus 2.5 MAD, and vice versa for data points below the median. This approach is similar to using 2.5 standard deviations from the mean, but is more robust by accounting for uncertainties on the measurements and because the estimates of the median and MAD are relatively insensitive to outliers. None of the paired measurement in this study that passed the first two criteria were classified as outliers.

## Results and Discussion

### A power law model for the flow-pressure relationship

**Fig 3:**
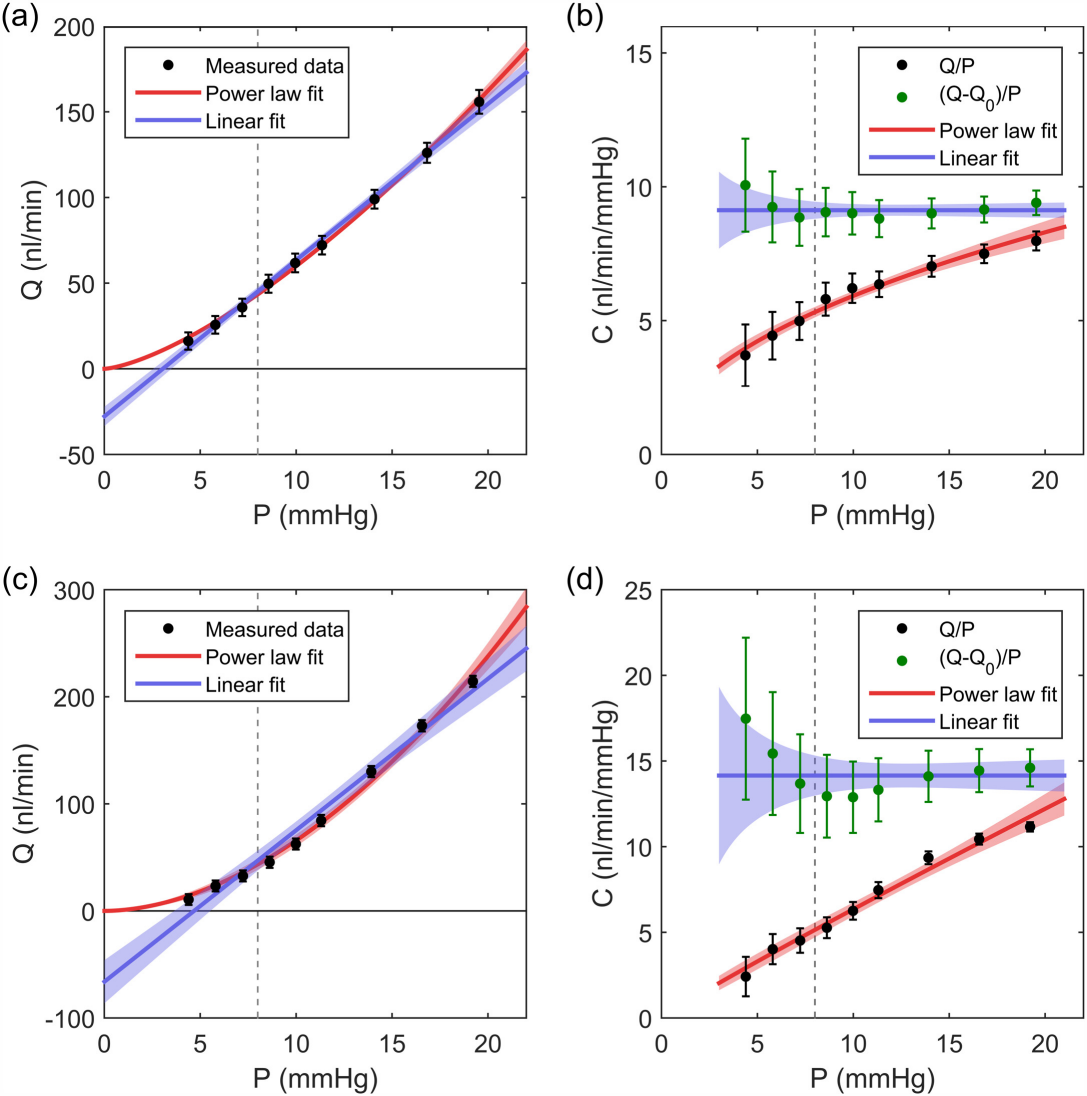
Selecting an appropriate model for the flow-pressure relationship. (a) A sample flow-pressure curve for the enucleated mouse eye perfusion shown in Fig 1b and 1c. Points show measured data with 95% confidence intervals. Blue: linear fit (Eq 3), *C* = 9.1 *nl*/*min*/*mmHg*, *Q*_0_ = −27.8 *nl*/*min*. Red: power law (Eq 9), *C*_*r*_ = 5.4 *nl*/*min*/*mmHg*, *β* = 0.44. Shaded regions show 95% confidence bounds. (b) The facility as calculated by the linear and power law models. Black markers show *Q*/*P*, which is independent of the fit, and green markers show facility as calculated according to Eq 8, (*Q* − *Q*_0_)/*P*, showing the large influence of the model on the calculated facility. (c) and (d) show equivalent plots for a more non-linear case. Linear fit, *C* = 14.2 *nl*/*min*/*mmHg*, *Q*_0_ = −66.2 *nl*/*min*. Power law, *C*_*r*_ = 5.4 *nl*/*min*/*mmHg*, *β* = 0.85.

Fig 3a shows a sample flow vs. pressure plot (for the same perfusion as the traces shown in Fig 1b and 1c). The linear fit to the data (Eq 6) with 95% confidence bounds are shown in blue. The raw data, shown in black, appear to be fairly well represented by the linear fit, aside from a small systematic deviation. The slope of the line, *C*_lin_ predicts a facility for this eye of 9.1 ^×^/ 1.06 *nl*/*min*/*mmHg*. The other free-parameter in the model, *Q*_0_, yields a value of −27.8 ± 5.7 *nl*/*min*. Considering Eq 3, the value of *Q*_0_ implies that if the pressure in the eye were zero, fluid would leave the eye at a rate of 27.8 *nl*/*min*. For an *in vivo* eye, some reverse flow would be expected due to AH secretion, but AH secretion is expected to be negligible in an enucleated eye.

In some outflow studies [12, 25], a non-zero value of *Q*_0_ was interpreted as ‘unconventional’ or pressure-independent outflow. In an enucleated eye, however, there is no clear physical mechanism that could generate a pressure-independent outflow. The osmotic driving force for uveovortex outflow [38], typically associated with pressure-independent outflow, would be eliminated by severing the ocular blood vessels during enucleation. We thereby posit that the term *Q*_0_ should be identically zero in enucleated eyes. In order to test this hypothesis, we measured the flow rate at an applied pressure of 0 *mmHg* in six enucleated mouse eyes, following a standard perfusion. For intraocular pressures of 0.036 ± 0.068 *mmHg*, the average flow rate was 1.1 ± 6.5 *nl*/*min* (WAM ± 2WSD). These values are negligibly different from zero. We thus propose that any flow-pressure model for enucleated mouse eyes should not include a *Q*_0_ term, such that there is no flow at zero pressure. As a corollary, including a non-zero *Q*_0_ term in Eq 3 may introduce errors in the estimation of facility.

To investigate the influence of a non-zero *Q*_0_ on the estimation of facility, consider Eq 3 rearranged in terms of facility at the *j*^*th*^ pressure step.

$$C_{\text{lin},j}=\frac{\left(Q_{j}-Q_{0}\right)}{P_{j}}$$ (8)

The values of *C*_lin,*j*_ are represented by the green data points in Fig 3b, with the blue line representing the constant value of *C*_lin_ as predicted by fitting a linear model to the data. Shaded regions represent the 95% confidence interval on the fit. However, as *Q*_0_ = 0 for enucleated mouse eyes, the facility for the *j*^*th*^ pressure step is not given by Eq 8 but rather by *C*_*j*_ = *Q*_*j*_/*P*_*j*_, as indicated by the black data points in Fig 3b. At a physiological pressure drop across the outflow pathway of 8 *mmHg* (represented by the vertical dashed lines), the facility predicted when including a non-zero *Q*_0_ value is almost twice that predicted when *Q*_0_ = 0, demonstrating that the estimation of facility is highly sensitive to assumptions regarding *Q*_0_. As illustrated in Fig 3c and 1d, the relative influence of *Q*_0_ on the estimate of facility is increased for more non-linear cases. Note that the absence of a *Q*_0_ term in enucleated eyes does not negate the presence of unconventional (non-trabecular) outflow, but does require that any unconventional outflow that exists be pressure dependent.

We thus require an alternative to the linear model, which allows both a pressure-dependent facility and a zero-intercept. We propose a simple power law model:

$$Q\;\left(P\right)=C_{r}\;{\left(\frac{P}{P_{r}}\right)}^{\beta }P$$ (9)

where *P*_*r*_ is a reference pressure (defined to be 8 *mmHg*), at which *C*_*r*_ is the facility. Following Eq 6, the model actually used to fit the measured data is

$$Q\;\left(P\right)=e^{Y_{r}}\;{\left(\frac{P}{P_{r}}\right)}^{\beta }P$$ (10)

where *Y*_*r*_ = log(*C*_*r*_). This fit is shown in red in Fig 3. The facility is given by

$$C\;\left(P\right)=C_{r}\;{\left(\frac{P}{P_{r}}\right)}^{\beta }$$ (11)

The power exponent *β* characterises the non-linearity of the flow-pressure relationship and can be interpreted as an index of the combined sources of non-linearity affecting the flow-pressure relationship through the outflow pathway. When *β* = 0, the facility is independent of pressure (*C* = *C*_*r*_ for all *P*) and the flow-pressure relationship is entirely linear. For *β* < 0, the facility decreases with increasing pressure, as may occur due to collapse of Schlemm’s canal, which increases outflow resistance with increasing IOP [39, 40]. For *β* > 0, the facility increases with increasing pressure. Evidence suggests that ‘anterior chamber deepening’ may artificially increase outflow facility as IOP increases by applying traction to the trabecular meshwork [41]. Anterior chamber deepening typically occurs during ocular perfusion via the anterior chamber, whenever the pressure in the anterior chamber exceeds that in the posterior chamber [42].

For the case shown in Fig 3a and 3b, *C*_*r*_ = 5.4 ^×^/ 1.04 *nl*/*min*/*mmHg* and *β* = 0.44 ± 0.06, which represents a modest non-linearity. Recall that for this case the linear model predicted *C*_lin_ = 9.1 ^×^/ 1.06 *nl*/*min*/*mmHg*. For the more non-linear case shown in Fig 3c and 3d, *β* = 0.85 ± 0.16 and *C*_*r*_ = 5.4 ^×^/ 1.12 *nl*/*min*/*mmHg*. For this case, the linear model predicts *C*_lin_ = 14.2 ^×^/ 1.13 *nl*/*min*/*mmHg*, which is almost three times the facility estimated by the power law model at physiological pressure drop.

In summary, our data reveal that the flow-pressure relationship is non-linear in enucleated mouse eyes and can be represented by a power law model that captures the pressure-dependence of outflow facility. Ignoring this non-linearity and fitting a linear flow-pressure relationship to the data introduces an error in the estimation of outflow facility, even when the non-linearity appears rather subtle as in the case of Fig 3a. This underscores the importance of specifying an appropriate model to interpret flow-pressure data and to estimate outflow facility.

### Comparison of regression parameters for the power law and linear models

In order to assess the power-law model and compare it to the commonly used linear model over a population of eyes, we evaluated the regression parameters from 66 independent, untreated eyes perfused with DBG. For each eye, the data were fit to both the linear and power law models.

**Fig 4:**
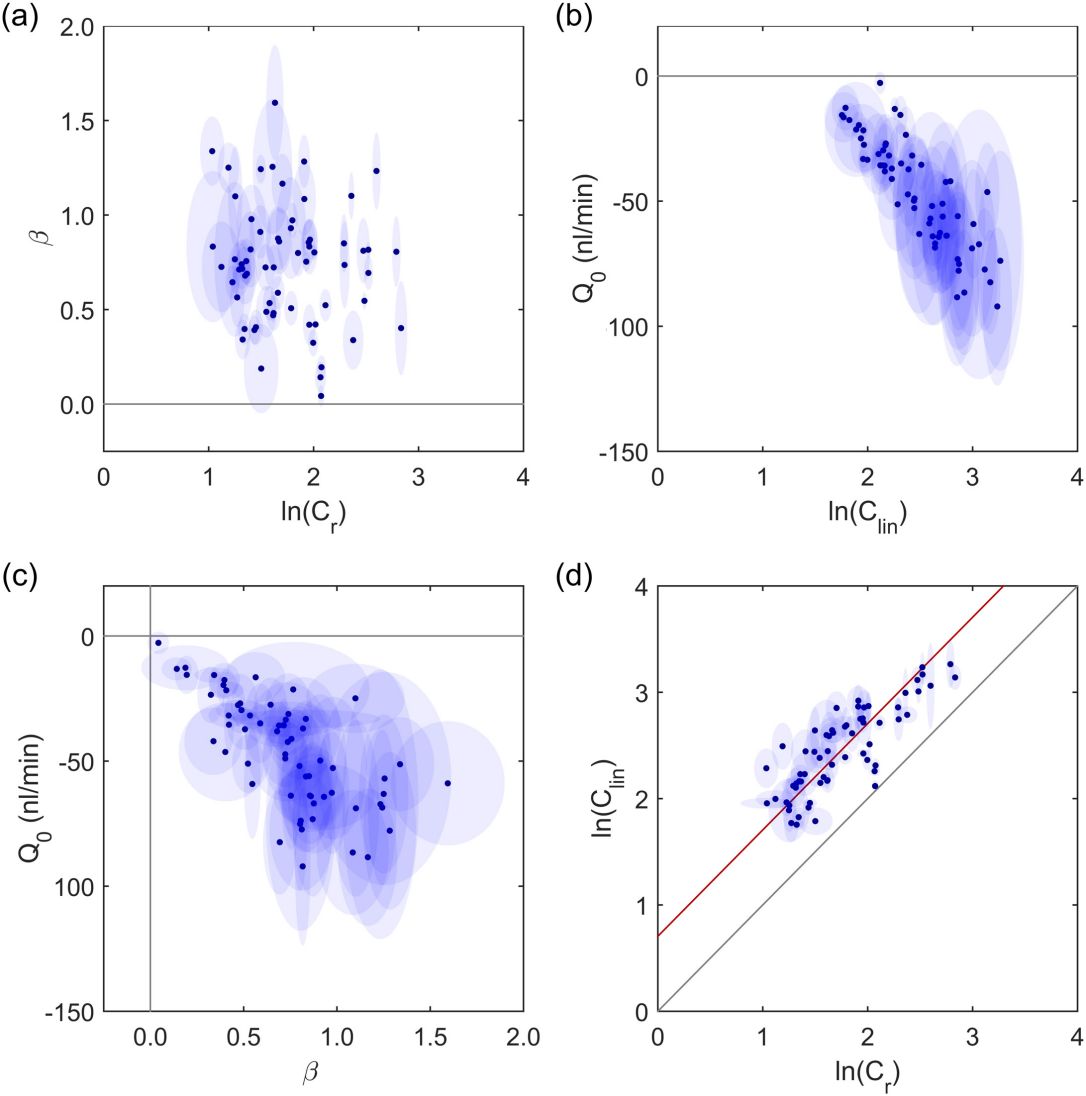
Comparing linear and power-law models. Outer ellipses show 95% CI on each parameter. (a) Power law exponent, *β*, against the logarithm of reference facility for the power law model: no correlation is observed (*p* = 0.49). (b) Pressure independent flow, *Q*_0_, against the logarithm of facility for the linear fit: a strong correlation is observed (*p* < 10^−6^), suggesting that the linear model is inappropriate. (c) Pressure-independent flow against power law exponent: a strong correlation is observed: *p* < 10^−6^, indicating that non-zero *Q*_0_ values are a result of the non-linearity in the *Q* − *P* relationship. (d) Comparison between log facility predicted by linear and power law models. Red line shows average over-prediction of ≈103% by the linear model.

As shown in Fig 4a, no correlation was observed between *β* and ln(*C*_*r*_), indicating that the regression parameters were independent of one another for the power law model. For the linear model, however, there was a strong correlation between ln(*C*_lin_) and *Q*_0_ (*p* < 10^−6^; Fig 4b), where larger negative values of *Q*_0_ coincide with larger positive values of ln(*C*_lin_). Based on current understanding of AH dynamics, there is no physiological precedent for a relationship between pressure-independent outflow and outflow facility, suggesting that the observed correlation is a result of insufficiencies in the linear model, rather than a physiological link between the two parameters. Similarly, there is no reason to suspect a relationship between *β* and ln(*C*_*r*_), and no such relationship was observed using the power law model (Fig 4a).

Fig 4c compares *Q*_0_ to *β*. When the non-linearity of the flow-pressure relationship was negligible (small *β*), so was the *Q*_0_ value, whereas for highly non-linear cases (large *β*), larger values of *Q*_0_ were found (*p* < 10^−6^). This suggests that the non-linearity of the flow-pressure relationship introduces an artificial non-zero value of *Q*_0_, as demonstrated by the data in Fig 3a and 3c.

As *Q*_0_ was negative in all cases, outflow facility as predicted by the linear model, *C*_lin_ was consistently overestimated. Fig 4d compares the facility calculated by the two models, showing that the linear model overestimated the facility with an average increase of ≈103%, as indicated by the red line, with a maximum of 268%. The error appears to be relatively systematic, implying that in studies using the linear model, changes in facility are still measurable; however, the scatter in the data implies that a larger number of samples would be required to achieve sufficient statistical power, compared to the power law model.

### Population distributions

**Fig 5:**
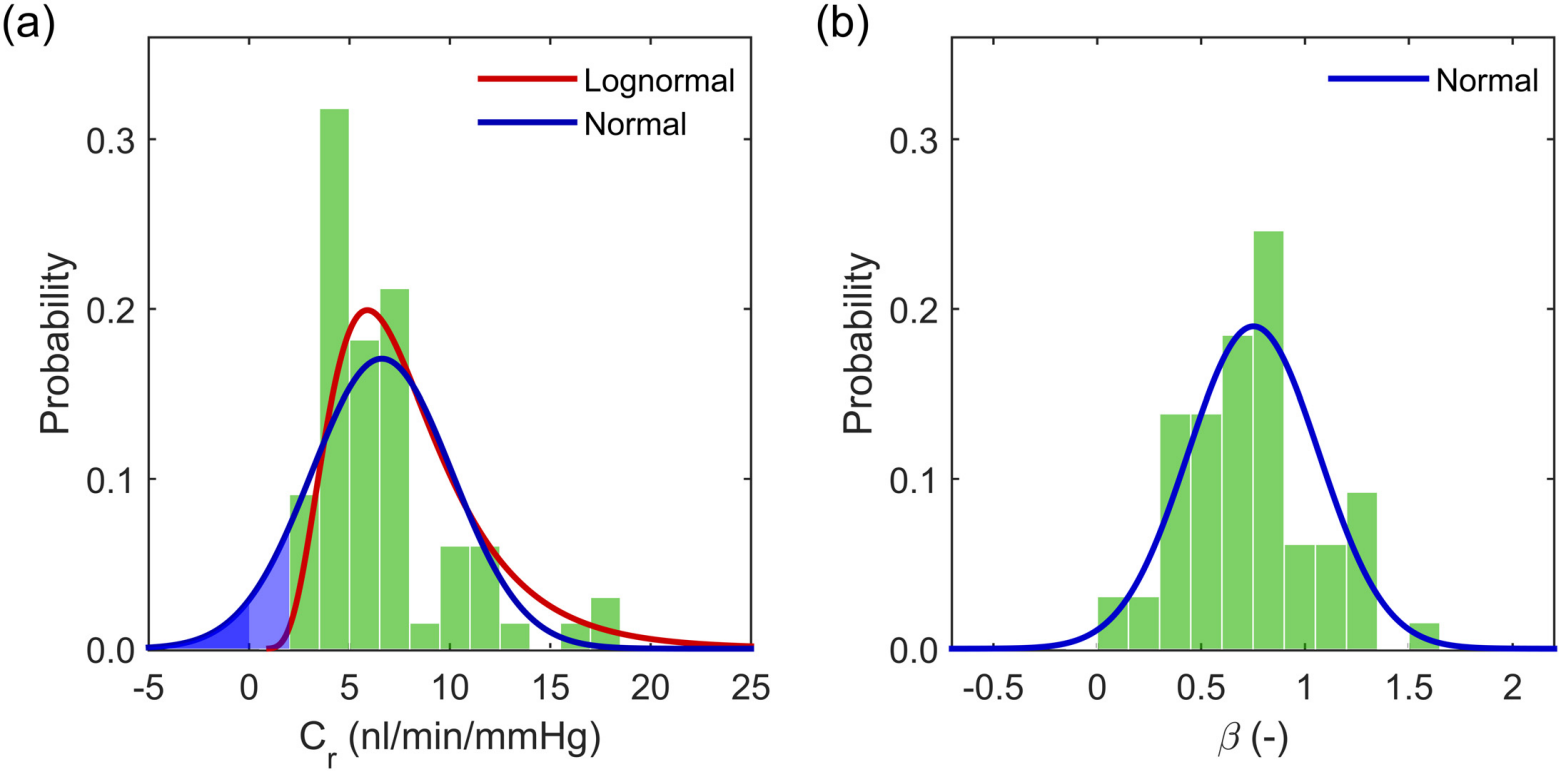
Population distributions of the regression parameters for the power law model. (a) Reference facility *C*_*r*_: histogram of 66 eyes with overlaid normal (blue) and lognormal (red) distributions. Shaded blue regions show predicted facilities below 2 *nl*/*min*/*mmHg* and below 0 *nl*/*min*/*mmHg*. (b) *β*: histogram from 66 eyes with overlaid normal distribution.

In this section, the population characteristics of the 66 independent B6 mice are investigated. Fig 5a shows a histogram of the *C*_*r*_ values. The histogram is skewed towards lower values of *C*_*r*_, although no mice had a facility below 2.5 *nl*/*min*/*mmHg*, and a number of mice exhibited relatively high facilities. Under the assumption of a lognormal distribution, the reference facility is best described in terms of the geometric mean and geometric standard deviation, yielding *C*_*r*_ = 5.89 ^×^/ 1.12(2.50) *nl*/*min*/*mmHg* ($\text{mean}\;{}^{\times }/\;ME_{95}(s_{\text{pop}}^{*2})$). Thus approximately 95% of the eyes measured lie in the range 2.4–14.8 *nl*/*min*/*mmHg*. The red curve in Fig 5a represents the predicted population distribution based on these values. As shown in the histogram, 3 eyes did have facilities greater than the upper confidence bound, which for 66 eyes could be expected. At the lower end of the scale, the distribution rapidly tails off and predicts that very few eyes would yield facilities below 2 *nl*/*min*/*mmHg* (1 in 109). Using the common assumption of normally distributed facilities, the population distribution would be described by *C*_*r*_ = 6.61 ± 0.85(7.01) *nl*/*min*/*mmHg*, as shown in blue in Fig 5a. The normal distribution predicts facilities of less than 2 *nl*/*min*/*mmHg* in 1 out of 11 mice, and negative facilities in 1 out of 34 B6 mice, as indicated by the blue shaded regions in Fig 5a. As negative facilities cannot exist, and the very low predicted facilities are not in agreement with the current measurements, *C*_*r*_ cannot be well described by a normal distribution.

In order to quantify how well the alternative distributions represent the data, we used the Shapiro-Wilk test [43], which evaluates the probability that null hypothesis that the data are sampled from a normal distribution is true for the observed data. Small *p*-values thus indicate that the data are unlikely to be sampled from a normal distribution. For the facility, *C*_*r*_, the Shapiro-Wilk test yields *p* = 2.5 × 10^−6^. For a lognormally distributed parameter such as *C*_*r*_, its logarithm *Y* = ln(*C*_*r*_) should be normally distributed. The Shapiro-Wilk test for ln(*C*_*r*_) yielded *p* = 0.016. The Anderson-Darling test (reported to be the second most powerful normality test after the Shapiro-Wilk test [43]) yielded similar results. Although the tests both reject the normality of the ln(*C*_*r*_) distribution at the standard threshold of *p* = 0.05, the *p*-value for ln(*C*_*r*_) is significantly larger than for the distribution of *C*_*r*_, indicating that the lognormal distribution is a considerable improvement.

The predicted population range in facility is rather large, considering that the mice should be genetically identical and their environment relatively constant. In order to investigate whether external factors contributed to the spread of *C*_*r*_, we examined the relationship between *C*_*r*_ and a number of possible confounding variables using Pearson’s correlation coefficient, *r*. The time of enucleation did not correlate with *C*_*r*_ (*r* = 0.014), suggesting that diurnal oscillations do not appear to be a cause of the large range in baseline facilities for the population. The time between enucleation and cannulation (*r* = −0.036) and the age of the mouse (*r* = 0.067) also showed no correlation with the measured facility. No effect of the day of the week on which the perfusion was carried out was detected using a one-way ANOVA (*p* = 0.10).

For the non-linearity parameter, *β*, the population distribution is shown in Fig 5b. In this case, the data are reasonably well represented by the normal distribution. The Shapiro-Wilk and Anderson-Darling tests yielded *p* = 0.044 and *p* = 0.086 respectively, indicating that it is reasonable to assume a normal distribution for this parameter. The distribution of *β* is described by *β* = 0.752 ± 0.079(0.631).

### Analysis of paired measurements

#### Reference facility

The large range of facilities observed in the population highlights the benefits of using paired analysis, which considerably reduces the parameter space by providing a contralateral control eye for each treated eye. In paired analyses, it is often tacitly assumed that if no modification was applied, then the control and experimental cases would yield identical values; that is the pairs are ‘ideal’. However, with such difficult measurements, and particularly with biological tissue, there will always be an inherent degree of uncertainty, and thus the extent of the ‘pairedness’ should be considered. In order to analyse the intra-individual variability between untreated contralateral eyes, *Ψ*_con_ = 10 pairs of eyes were perfused simultaneously with DBG on duplicate systems.

**Fig 6:**
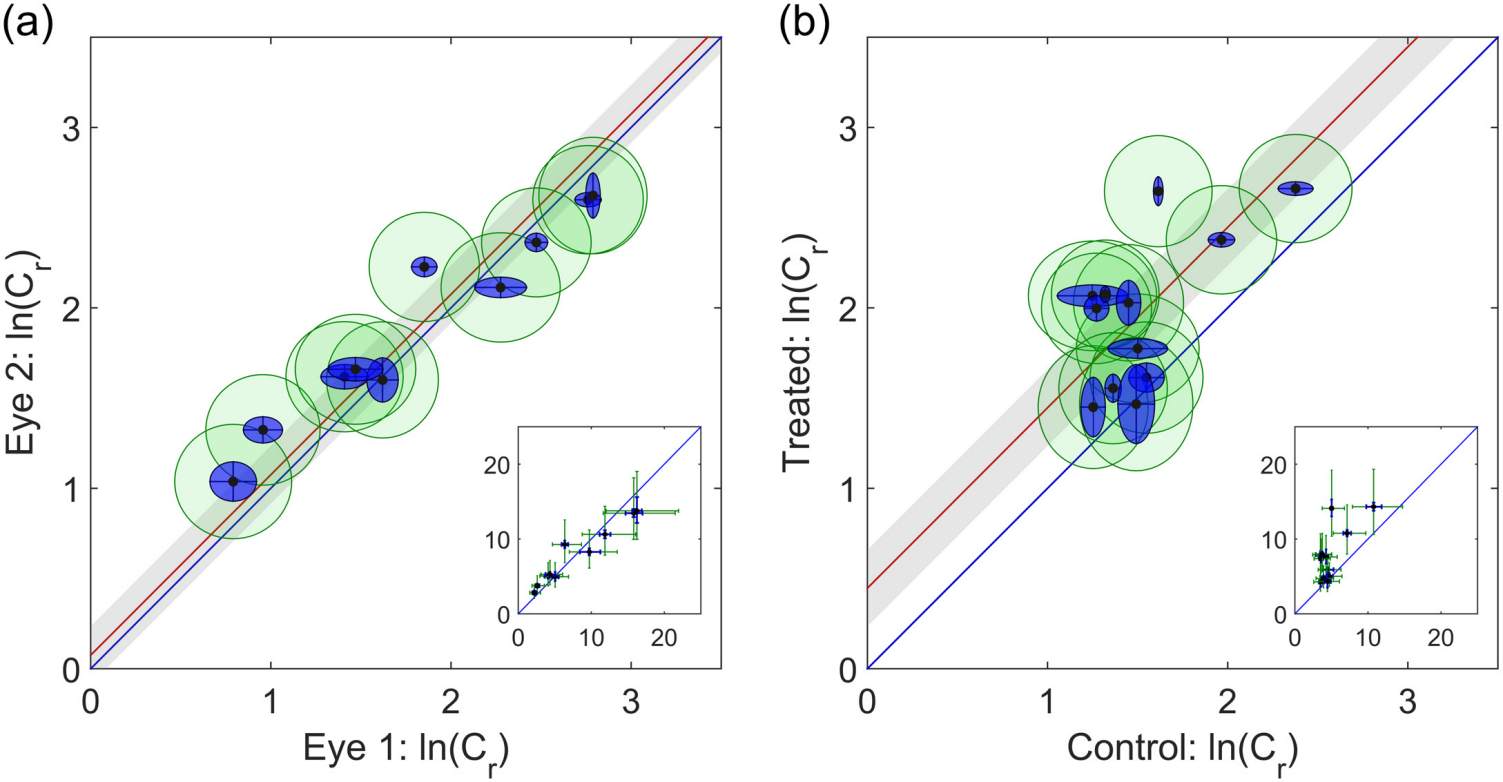
Comparison of facility for paired eyes using ‘Paired Facility plots’. Each data point represents one individual, with the log-transformed facility of each eye defining the co-ordinates. Filled ellipses indicate 95% confidence intervals from the regression fitting of Eq 9 (1.96*s*_reg_) and outer ellipses indicate additional uncertainty due to intra-individual variability (*s*_con_). Inset: facility in *nl*/*min*/*mmHg*. Unity line is shown in blue. Red line shows average difference $\bar{Z}$, with its confidence intervals in grey. (a) Untreated contralateral pairs. No pairs exhibited a significant difference between contralateral eyes when accounting for uncertainty. (b) Effect of PDA205 treatment compared to control eyes. A significant increase in the facility following treatment is observed, although there is considerable variability ($s_{\text{tre}}^{2}$) introduced by the drug.

For each pair, *p*, the value of *Z*_*p*_ (the difference in the log-transformed facility between the treated and control eye) was calculated. Fig 6a shows a ‘paired facility plot’ for the data. The *x* and *y*-axes are the log-transformed facility of the two contralateral eyes, therefore each pair yields a single point on the graph, with the blue ellipses indicating the 95% confidence intervals based on the output of the weighted regression fit of Eq 9. It can be seen that, even when accounting for the confidence intervals from the regression, not all data points overlap the unity line (shown in blue), which would indicate a measurement that is equal for both untreated eyes. Therefore, an additional source of uncertainty must exist, which can be attributed to the intra-individual variability, $s_{\text{con}}^{2}$.

In order to estimate $s_{\text{con}}^{2}$, we assume that the total deviations from the unity line, $s_{\text{dif}}^{2}$, result from both the intra-individual variability and the regression uncertainty, such that $s_{\text{dif}}^{2}=s_{\text{con}}^{2}+2\;\bar{s_{\text{reg}}^{2}}$. The value of $s_{\text{dif}}^{2}$ is estimated using the mean squared difference, which would be equivalent to the unbiased variance of *Z*_*p*_, if $\bar{Z}$ were zero. Using the average $\bar{s_{\text{reg}}^{2}}$, we can estimate $s_{\text{con}}^{2}$ according to

$$s_{\text{con}}^{2}=\frac{1}{\Psi_{\text{con}}}\sum_{p=1}^{\Psi_{\text{con}}}Z_{p}^{2}-2\;\bar{s_{\text{reg}}^{2}}$$ (12)

Please see S2 File for further details. For the B6 mice analysed here, $s_{\text{con}}^{2}=0.046$, giving two-sigma of 2*s*_con_ = 0.430, as a difference in the log domain. This corresponds to a 53% increase or a 35% decrease in facility, for a given pair. The green outer ellipses in Fig 6a show the uncertainty including *s*_con_. For each of the pairs, the outer ellipses cross the unity line, and thus the facilities for the two eyes from each mouse are not significantly different from one another when accounting for intra-individual variability. Analysing these data in terms of the difference in log-transformed facility between contralateral eyes yields $\bar{Z}=0.075\pm 0.160$, which is not significantly different from zero. The red line in the figure shows $\bar{Z}$, and the grey shaded area shows the confidence interval on $\bar{Z}$ ($\bar{Z}\pm ME_{\bar{Z},95}$), which overlaps the blue unity line, and thus $\bar{Z}$ is not statistically different from zero. See Table 1 for a summary of the key statistical parameters.

**Table 1:** Summary of the results of the untreated and PDA205 analyses. All terms are dimensionless. $p\;(\bar{Z}=0)$ is the probability that the observed $\bar{Z}$ is not significantly different from zero, according to the weighted *t*-test described in S2 File.

Case	$\bar{Z}$	$ME_{\bar{Z},95}$	2*s*_tre_	%	${\bar{D}}^{*}$	$ME_{{\bar{D}}^{*},95}$	$s_{\text{tre}}^{*2}$	$p(\bar{Z}=0)$
Untreated Pairs	0.075	0.160	-	8	1.08	1.17	-	0.3159
PDA205 Paired	0.446	0.212	0.487	56	1.56	1.24	1.63	0.0007
PDA205 Unpaired	0.440	0.322	0.543	55	1.55	1.38	1.72	0.0100

Although variability between contralateral eyes appears to be fairly large, it is still possible to use the system to conclusively measure differences in facility. To verify this, 12 pairs of eyes were perfused intracamerally with 10 *nM* 3,7-dithia prostaglandin E_1_ (PDA205) or vehicle control in DBG. PDA205 (Allergan Inc., Irvine CA) was stored as a lyophilised powder at −80°*C*, dissolved in ethanol as a 10 *mM* stock solution, stored at −20°*C* and protected from light at all times. Working stock was made up daily by diluting the stock solution in DBG. The control eye received DBG with an equivalent concentration of vehicle (17 *μM* ethanol). PDA205 is an EP_4_ agonist and has been shown previously to increase outflow facility in enucleated mouse eyes [11], enucleated human eyes [44] and living monkey eyes [45].

The results of the PDA205 experiments are presented as a paired facility plot in Fig 6b. The *x*-axis is the log-transformed facility of the control eyes, and the *y*-axis is the log-transformed facility of the treated eyes. For data points lying above the blue unity line, an increased facility was measured in response to the treatment. Although PDA205 is known to increase outflow facility, of the 12 pairs examined here, only 5 pairs independently exhibited a significant difference in facility in response to treatment (green ellipses did not cross the unity line). However, all pairs did show some increase in facility, and thus a statistically significant increase was observed overall. For these data, $\bar{Z}=0.446\pm 0.212(0.487)$. This gives a proportional change of ${\bar{D}}^{*}=1.56\;{}^{\times }/\;1.24(1.63)$. These data imply an overall percentage increase in facility of 56% in response to PDA205 (*p* = 0.0008). The two-sigma on the percentage facility increase for individual pairs, however, spanned a wide range from -4% to 154%. One could thus conclude from these data that PDA205 increases outflow facility on average, but that the effect of the drug varies considerably between individuals.

#### Non-linearity parameter

For the 10 untreated pairs, the power law exponent, *β* was not correlated between paired eyes (*r* = 0.246). However, when one case in which the maximum pressure was less than 9 *mmHg* (and thus *β* may not be well characterised) was removed, the correlation coefficient increased to *r* = 0.799 (*p* = 0.0097), implying that the pressure-dependence of facility may be paired between contralateral eyes. Applying Eq 12 to the nine *β* values yielded a two-sigma of 2*s*_con,*β*_ = 0.322, approximately half the average value for the population.

### Analysis of unpaired measurements

**Fig 7:**
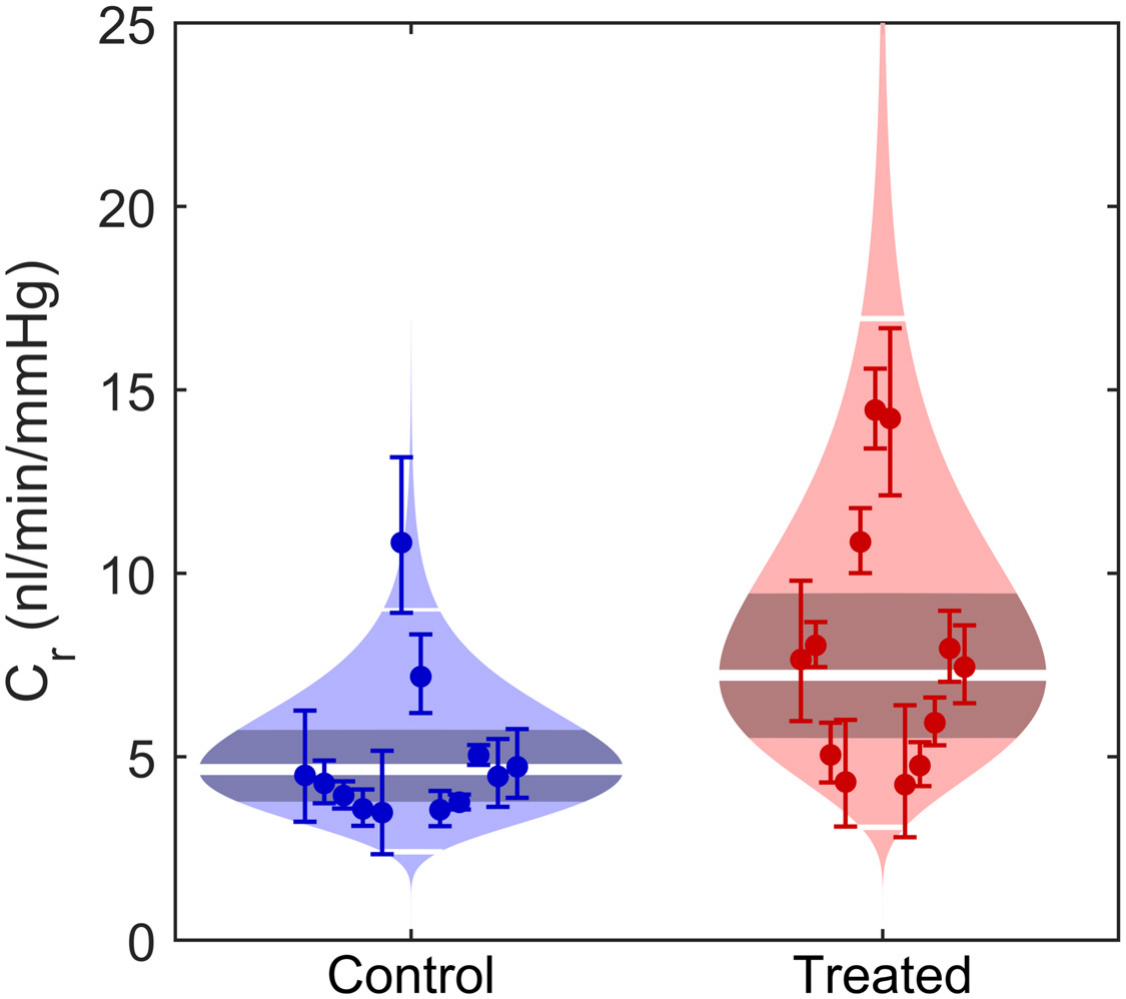
Comparison of facility for unpaired eyes using the ‘Cello plot’. Unpaired analysis of facility for PDA205 treated and control eyes. Each data point shows the reference facility, *C*_*r*_, with the error bars showing 95% confidence intervals from the regression fitting of Eq 10 (1.96*s*_reg_). Shaded regions show best estimates of the sample distributions, with the geometric mean and two-sigma shown by the thick and thin horizontal lines respectively. Dark central bands show 95% CI on the mean values.

In some cases, it is not possible to perform paired measurements between contralateral eyes. In order to demonstrate the processing for unpaired data, the PDA205 data set was also analysed as if the control and treated eyes were obtained from different individuals. Fig 7 shows a modified form of a violin plot of the data, which we refer to as a ‘cello’ plot. The individual *C*_*r*_ values for each eye are shown by the markers, with the 95% confidence intervals on the regression uncertainty shown by the error bars. As described above, there will be an additional uncertainty on *C*_*r*_ related to the cannulation, but we cannot quantify this uncertainty independent of the population variability, and it is therefore not indicated in the cello plot. The shaded region in the background indicates the predicted lognormal distribution of *C*_*r*_, as described by the calculated ${\bar{C_{r}}}^{*}$ and $s_{C_{r}}^{*}$, and the width is scaled such that area of each shaded region is equal for the control and treated eyes. The geometric mean ${\bar{C_{r}}}^{*}$ is indicated by the thick central white line, and the thin white lines indicate two-sigma, within which 95% of eyes from this population are expected to lie. The grey area in the centre of the data set indicates the 95% confidence interval on ${\bar{C_{r}}}^{*}$. As compared to a box plot, the cello plot shows all individual data points and their uncertainty, as well as the predicted distribution and the confidence interval on the mean, providing a complete overview of the data. Consistent with the paired analysis, the cello plot in Fig 7 shows an increase in *C*_*r*_ in response to treatment with PDA205 using unpaired analysis.

The statistics for the unpaired analysis yield a similar proportional increase in response to PDA205 to that calculated with the paired analysis, ${\bar{D}}^{*}=1.55$ (see Table 1). However, the CI on the mean proportional change increases significantly for the unpaired analysis, as evidenced by $ME_{{\bar{D}}^{*},95}$ increasing from 1.24 to 1.38. Correspondingly, the calculated *p*-value is more than an order of magnitude greater when the data were analysed as unpaired relative to paired, although both *p*-values would still be considered significant.

PDA205 has a relatively large facility effect, however, and thus treatments having smaller effect sizes may not achieve sufficient statistical power to be detected by unpaired analysis without using prohibitively large number of animals. Note also that the value of $s_{\text{tre}}^{*2}$ increases, but by relatively little, from 1.63 for paired analysis to 1.72 for unpaired analysis, indicating that both methods predict a similar variability in the treatment effect.

### Recommendations

The measurement of outflow facility in mice, or indeed any animal, is technically challenging. A number of different approaches have been reported in the literature for acquiring pressure and flow data during ocular perfusions, to which a model is fitted in order to estimate the facility. Subsequently, statistical analysis is carried out on the facilities of treated and control eyes, in order to establish whether observed trends are significant. Based on findings from this study, we make the following recommendations regarding measurement of outflow facility in enucleated mouse eyes.

#### System validation

In order to analyse the uncertainty, one must first establish that the pressure and flow can be measured with sufficient accuracy and resolution in the physiological range of interest. As the facility is the main parameter of interest, acceptable facility measurements should be demonstrated in the appropriate range, which can be done using glass capillaries. Similar validation would be necessary for techniques to measure other parameters in live animals, such as AH secretion, episcleral venous pressure or unconventional outflow.

#### Steady state condition

Due to the compliant response of the eye to a change in pressure or flow, an accurate measurement of facility can only be acquired when the system has reached steady state. An objective steady state condition should be defined and its implications for the measurement of facility should be discussed.

#### Pressure steps

The pressure steps used in the present protocol have been selected so as to provide high resolution around the physiological range of pressure drops across the outflow pathway (≈8 *mmHg*), as well as data at elevated intraocular pressures. Both the range and number of pressure steps are arbitrary and can be tailored to a particular experiment, but should be held constant as much as possible for comparing cases within a given experiment. Increasing the number of pressure steps improves confidence intervals on the facility estimate and thereby effectively increases measurement accuracy, but also increases the duration of the experiment.

#### Model fitting

The present results demonstrate that the flow-pressure relationship is non-linear in enucleated mouse eyes, and the standard linear fit (and assumption of a facility that is independent of pressure) is therefore not appropriate. Applying a linear fit leads to the appearance of pressure-independent outflow, but no such outflow is present in enucleated mouse eyes. Furthermore, using the linear model to estimate facility, even when the flow-pressure relationship appears relatively linear, may result in errors up to 250%. For these reasons, we recommend using the power law model proposed in Eq 9. We also advocate showing sample flow-pressure relationships, along with the fit and 95% confidence bounds, to demonstrate the quality of the perfusion data.

#### Statistical distribution

The present data have shown that facility is better described by a lognormal distribution than the commonly assumed normal distribution. In order to make use of the standard statistical tools that require normality, the log-transformed facility should be obtained by fitting Eq 10 to the flow-pressure data, and all statistical analysis should be conducted in the log domain.

#### Data analysis

Ocular perfusion measurements, particularly those in mouse eyes, include several sources of uncertainty that may vary between individual samples. Additionally, the average treatment size and variability in the treatment effect are of importance. For these reasons, we developed statistical tools, including the weighted *t*-test, which can be applied to any perfusion data, and introduced the paired XY plot and cello plot. Use of the data analysis methods reported here would improve the transparency of data in future studies and enable improved comparability between studies.

#### Negative results

It is not possible to conclude from an experiment that a given experimental condition has no effect. However, using the statistics developed in this paper, an appropriate CI on $\bar{Z}$ can be determined, from which one can obtain an estimate of the minimum effect size that could have been detected. For example, in the paired analysis of the PDA205 data set, $ME_{\bar{Z},95}=0.214$. This implies that, for the twelve pairs we measured, the minimum net change we would have been able to statistically resolve would be ±0.214, a fold change of approximately ±20%.

### Limitations

A number of outstanding issues remain in the development of methods to accurately measure outflow facility in mice. One issue is that, when using a single needle to perfuse with compounds via the anterior chamber, it is unclear whether the compound has acted on the entire outflow pathway before facility measurements are initiated. Considering an average B6 mouse with a facility of 6 *nl*/*min*/*mmmHg* at a physiological pressure drop of 8 *mmHg*, it would take more than two hours to turnover the anterior chamber volume, which is approximately 6 *μl*[21]. This problem is exacerbated by segmental outflow that may deliver the compound non-uniformly to the trabecular meshwork [46, 47]. Variability in the uniformity of drug delivery may elevate the apparent value of $s_{\text{tre}}^{2}$ and increase the number of mice required to achieve sufficient statistical power. A possible solution is to fully exchange the contents of the anterior chamber with the treatment solution prior to the start of the stepping protocol, but this would require a second perfusion needle, which is technically challenging and would likely introduce additional variability into the facility measurements. Alternatively, it may be possible to pre-treat the eyes prior to the perfusion either topically or intracamerally, assuming that this delivery did not interfere with the perfusion and was compatible with the time scale of the facility effect.

A second question is the mechanism of the pressure-dependent increase in outflow facility. In other species such as primates, outflow facility typically decreases with increasing pressure [40], which has been attributed to pressure-induced collapse of Schlemm’s canal [39]. If perfusions are carried out *ex vivo* via the anterior chamber, however, the resulting pressure difference generated across the iris exerts traction on the outflow pathway and increases outflow facility; this phenomenon is known as ‘anterior chamber deepening’ [48] and is an experimental artefact that can be overcome by perfusing via the posterior chamber or by creating a shunt across the iris (iridectomy) [41]. The eyes in this study were perfused via the anterior chamber without an iridectomy, and thus it is possible that anterior chamber deepening may have contributed to the pressure-dependent increase in outflow facility and to the non-linearity of the flow-pressure relationship. It is conceivable that pressure dependence in both the conventional and unconventional outflow pathways could contribute to the observed non-linearity. However, it is not possible to distinguish reliably between alternative anatomical routes based on perfusion data alone. Unconventional outflow is commonly regarded to be relatively pressure insensitive [22, 49] and therefore increasing unconventional outflow facility seems an unlikely candidate for the observed pressure-dependent increase in total outflow facility.

Finally, we accept that as the perfusions were conducted on post-mortem enucleated eyes, the current experiments do not fully capture the mechanisms that may influence outflow facility regulation *in vivo*. This includes the potential effect of innervation, vascular regulation or other local or systemic factors that may influence outflow facility. As discussed below, *in vivo* measurements have their own limitations regarding other aspects of AH dynamics that may change during the perfusion and thereby complicate measurements of facility in living animals, but some of these *in vivo* limitations may be overcome using the *iPerfusion* system. Studies to establish *in vivo* techniques using *iPerfusion* are currently underway.

### Implications for *in vivo* perfusions

The main advantage of perfusing enucleated eyes, relative to eyes maintained *in vivo* or *in situ*, is control over the parameter space. In enucleated eyes, the variables *Q*_*in*_, *P*_*e*_ and *Q*_0_ in Eq 1 are zero, and the temperature, hydration state of the eye and external pressure can be accurately controlled by fully immersing the eye in isotonic saline. Conversely, for *in vivo* experiments, *Q*_*in*_ and *Q*_0_ are unknown, the downstream pressure in the conventional outflow pathway, *P*_*e*_, is determined by episcleral venous pressure, and the hydration state of the eye is harder to control. Furthermore, anaesthesia may alter these parameters during a perfusion. For *in situ* measurements in euthanised animals, the values of *Q*_*in*_, *Q*_0_ and *P*_*e*_ would decay to zero eventually, but the time scales of this decay are unknown and would likely differ between parameters. Although a range of complementary methods may be used to estimate these variables, the compound uncertainties in each variable would weaken confidence on the estimates on the final parameters. These added complications can be justified by the fact that ultimately *in vivo* perfusions are a direct measurement of the entire system of ocular fluid dynamics, rather than a disconnected component as measured *ex vivo*. However, the present findings suggest that some of the common assumptions made in the analysis of *in vivo* data may not be appropriate.

In living animals, AH secretion, *Q*_*in*_, pressure-independent outflow, *Q*_0_, and episcleral venous pressure, *P*_*e*_, are all expected to be non-zero, and these terms would need to be added to an analogous form of Eq 9 that is appropriate for *in vivo* pefusions. If one assumed that *Q*_0_, *Q*_*in*_ and *P*_*e*_ were independent of both time and pressure, then a potential model for the flow-pressure relationship in living mouse eyes would be given by $Q\;(P)=C_{r}\;{\left(\frac{P-P_{e}}{P_{r}-P_{e}}\right)}^{\beta }\;(P-P_{e})+Q_{0}-Q_{in}$, where the total pressure-independent flow in the system is *Q*_0_ − *Q*_*in*_, and would be negative indicating a net flow from the eye into the perfusion system at *P* = *P*_*e*_. Due to the non-linearity of facility, *Q*_*in*_ − *Q*_0_ could not be accurately estimated by extrapolation, but would need to be measured directly when *P* = *P*_*e*_. Additionally, knowledge of *P*_*e*_ is necessary to estimate *C*, which would not be the case if *C* was independent of pressure. Methods have been reported to estimate *P*_*e*_ and *Q*_*in*_ in mouse eyes, but the accuracy of such measurements requires validation [12, 13, 21].

An additional and considerable complication in the context of *in vivo* perfusions is the change in IOP over time under anaesthesia [31, 50–52]. Episcleral venous pressure is directly correlated with mean arterial pressure [53] and will thus decrease over time, as well as being influenced by topical anaesthesia [54]. AH secretion, *Q*_*in*_, is also reduced under anaesthesia [55], despite its mechanism being predominantly independent of pressure. The change of these variables in time, introduces additional uncertainty in the estimation of outflow facility. The two-step constant pressure perfusion commonly used in monkeys to account for time-varying parameters inherently assumes a facility that is independent of pressure [24], and so may not be completely appropriate if *in vivo* mouse eyes exhibit a non-linear flow-pressure relationship, as observed *ex vivo*.

## Conclusions

In this study, we described *iPerfusion*, a constant pressure perfusion system that provides faster and more accurate measurements of outflow facility in enucleated mouse eyes, relative to perfusion systems previously described in the literature. We fully characterised the *iPerfusion* system in the physiological range of facility values relevant for mice, including *in vitro* tests and *ex vivo* studies. We developed a statistical analysis and the weighted *t*-test to account for uncertainties in the perfusion measurements and in the estimation of facility, and devised graphical formats to present the data in a manner that appropriately displays the uncertainty and spread in the measurements.

Using *iPerfusion*, we demonstrated that there is negligible pressure-independent outflow in fully hydrated enucleated eyes from young B6 mice. Using a 9-step perfusion protocol, we showed that the flow-pressure relationship was consistently non-linear and as a consequence, inappropriate application of a linear model to the data led to considerable overestimation of outflow facility. In contrast to the linear model, the flow-pressure relationship was well described by a simple power law model that captured the pressure-dependence of outflow facility. Comparing the linear and power law models revealed that the appearance of pressure-independent outflow when analysed using the linear model is most likely attributable to the non-linearity in the flow-pressure relationship and the inappropriate assumption of pressure-independent outflow facility.

Analysis of 66 mice revealed that baseline facility is better described by a lognormal distribution than a normal distribution. Hence, statistical analysis of facility should be carried out in the log domain, by using log-transformed values. The distribution of baseline facility values spanned nearly a 6-fold range, but facility was relatively tightly correlated between contralateral eyes. This large variability between individuals was not attributable to any apparent experimental condition, and given the correlation between contralateral eyes, it appears that this spread may have a physiological origin. As IOP variability within the B6 population has been reported to be 14.6 ± 5.2 (mean ± 2SD) [56], such a large spread in facility is unexpected and may suggest that facility is somehow regulated to maintain a fairly constant IOP, despite other systemic variables that may affect AH dynamics. Further research is needed to investigate the basis of the wide distribution of facility between individuals, but the fact that the spread is so large emphasises the advantage of using a paired experimental design.

With minor modifications, the *iPerfusion* system may be translated to other species and to *in vivo* ocular perfusion, as well as perfusion of other biofluidic systems.

## Supporting Information

S1 File Supporting Information 1: Analysis of Sensors. An analysis of the accuracy and repeatability of the pressure and flow sensors used in the *iPerfusion* system.
(PDF)

S2 File Supporting Information 2: Statistical Analysis. A description of the statistical methodology developed for use with *iPerfusion*, which takes into account the uncertainties in the measurements and includes the ‘weighted *t*-test’.
(PDF)

S3 File Supporting Information 3: Nomenclature. Summary of the mathematical notation used in the main text.
(PDF)

S4 File Supporting Information 4: Raw Data. The raw data from the perfusion traces along with the steady state values for each pressure step.
(ZIP)
