# Supplementary material for: Measurement of Outflow Facility Using iPerfusion
Source: PLoS One. 2016 Mar 7;11(3):e0150694. doi: 10.1371/journal.pone.0150694 (PMC4780770; doi:10.1371/journal.pone.0150694)
Supplement: S1 File — An analysis of the accuracy and repeatability of the pressure and flow sensors used in the iPerfusion system. (PDF) [file pone.0150694.s001.pdf]

Measurement of Outflow Facility using *iPerfusion*

Joseph M. Sherwood, Ester Reina-Torres, Jacques Bertrand, Barnaby Rowe, Darryl R. Overby

Supporting Information 1: Analysis of Sensors

This Supporting Information provides an analysis of the accuracy and repeatability of the pressure and flow sensors used in the *iPerfusion* system. For each measurement, components of uncertainty arising due to the sensor itself and from averaging a noisy signal over a finite time period will be considered.

S2-1 Analysing Uncertainty

Every measurement has associated with it some error, which results in the measurement deviating from the true value. Given this limitation, it is only possible to state the true value using the measured value M_{sens} with some degree of uncertainty

$$M_{\text{true}} = M_{\text{sens}} \pm s_{\text{sens}} \tag{S1-1}$$

In order to characterise the uncertainty in the sensor, s_{sens}, one must devise an experiment wherein the true value can be defined, and then analyse the deviation between the sensor measurement and the true value.

In the *iPerfusion* system, the height of the pressure reservoir is controlled using a linear actuator that has a stepper-motor with a microstep size of $1.25\mu m$. A single microstep would therefore correspond to a pressure change of $0.00009\ mmHg$. In practice, although imperfect alignment of the spindle would likely add some variability, it seems reasonable to expect that the error between the specified and actual height would deviate by less than 10 microsteps. We therefore assume that the applied pressure can be controlled with an accuracy exceeding $0.001\ mmHg$. The applied pressure from the reservoir for a given pressure step j, $P_{a,j}$, is thus considered to be the true pressure, and P_a is used to determine the uncertainty in both the pressure and the flow sensors, as described below.

S2-2 Pressure Sensor Uncertainty

For the custom Omegadyne PX409 used in the *iPerfusion* system, the accuracy is reported to be 0.08% of the full scale range of $0 - 50\ mmHg$, corresponding to $0.04\ mmHg$. Here, we characterise the uncertainty in the pressure sensor within the context of the *iPerfusion* system.

The pressure sensors are calibrated for each day of use and these data can be used for analysis of sensor uncertainty. The following process is used for calibrating the pressure sensors. Firstly, the pressure on both sides of the sensor is set to be exactly equal, corresponding to zero

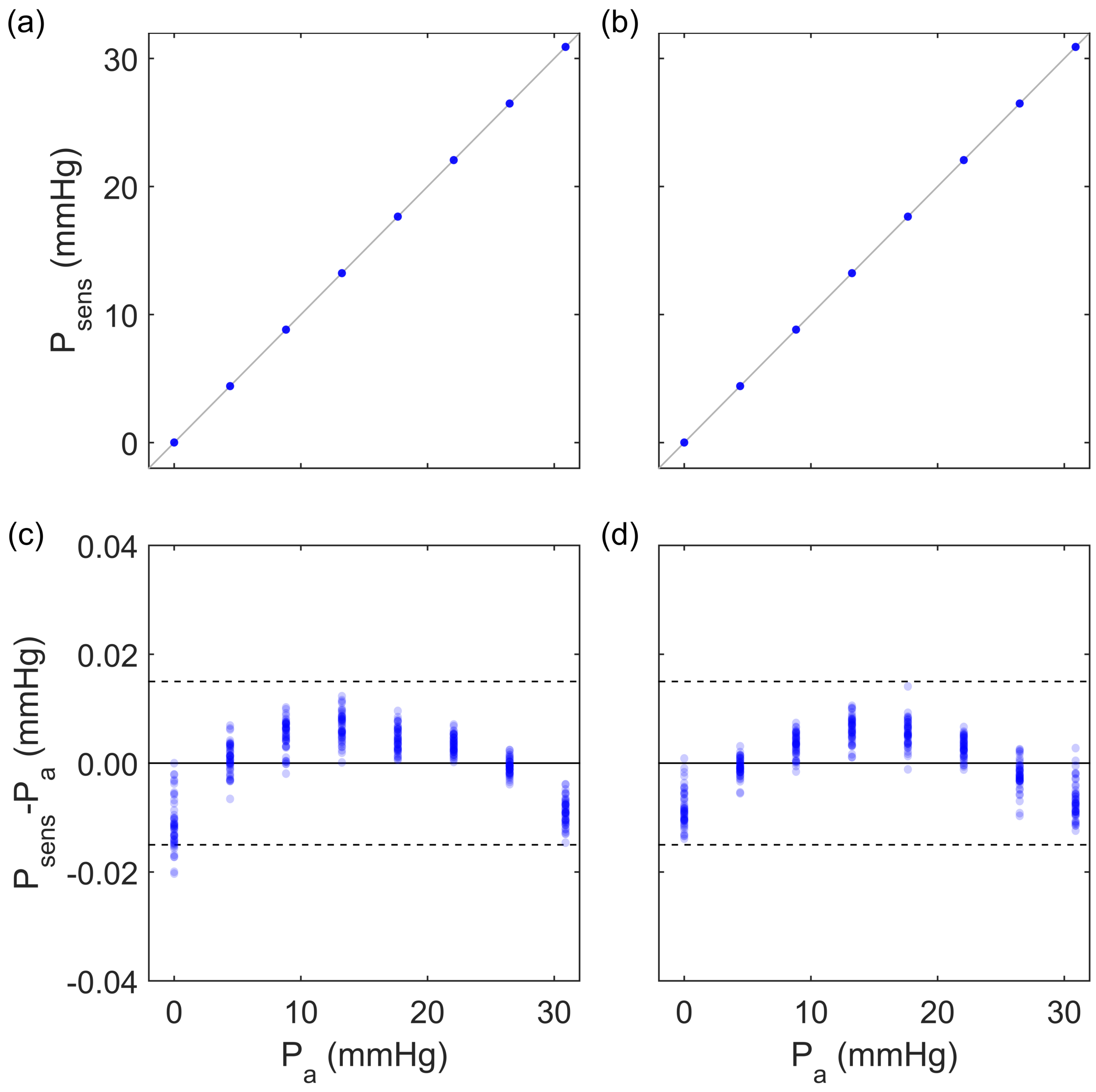

Figure S1-1: Analysis of the pressure sensors. Results of 50 calibrations over a year for the two pressure sensors used in the duplicate *iPerfusion* systems. (a) and (b) show the measured pressure against the applied pressure, P_a, for all 50 calibrations. (c) and (d) show residuals from the straight line fit, comparing the difference between the measured and applied pressure. Dashed lines indicate maximum uncertainty calculated according to Equation (S1-4).

pressure difference, by opening both sides to the same reservoir and recording the voltage output from the pressure sensor. One side of the pressure sensor is then opened to the actuated pressure reservoir, and the control software programmatically locates the height that gives the voltage recorded in the first step, which by definition corresponds to zero pressure. An 8-step automated calibration is then carried out, increasing P_a in increments of $60\ mm$, and averag-

ing the voltage over 5 seconds at $1000\ Hz$ for each step (in order to minimise the influence of electrical noise). A linear fit is then applied to the voltage-pressure relationship

$$V = V_p\,P_a + V_0 \tag{S1-2}$$

This straight line fit yields parameters V_p and V_0, which can be used to calculate the measured pressure

$$P_{\text{sens}} = \frac{(V - V_0)}{V_p} \tag{S1-3}$$

In order to estimate $s_{P_{\text{sens}}}$, we analyse the residuals between the measured and applied pressure. To perform this analysis, we consider 50 calibrations selected randomly and taken over a period of more than a year. Figures S1-1a and b show the measured pressures calculated using Equation S1-3 against the applied pressures, with the two panels showing two different sensors of the same model used in our duplicate systems. The 50 data points collapse upon one another, such that all calibrations appear identical. Figures S1-1c and d show the residuals from the straight line fit, $P_{\text{sens}} - P_a$.

Both sensors clearly have a very repeatable non-linearity, which persisted over numerous measurements over a long period of time. For each of the 50 calibrations, the uncertainty $s_{P_{\text{sens}}}$ is given by

$$s_{P_{\text{sens}}} = \sqrt{\frac{\sum_{i=1}^{N} (P_{\text{sens}} - P_a)^2}{N - 2}} \tag{S1-4}$$

where N is the number of pressure steps, and $N - 2$ is the number of degrees of freedom in the linear regression. Over the 100 calibrations using both sensors, the median value of $s_{P_{\text{sens}}}$ was $0.007\ mmHg$, with a maximum value of $0.012\ mmHg$. The maximum $s_{P_{\text{sens}}}$ value is used as a conservative estimate of the standard deviation in pressure attributable to sensor uncertainty, and is indicated by the dotted lines in Figures S1-1c and d.

S2-3 Flow Sensor Uncertainty

The flow sensor used in this study was the Sensirion SLG64-0075. This sensor has a range of $\pm 5000\, nl/min$, and an accuracy reported by the manufacturer of 10% of the measured value for flow rates greater than $250\ nl/min$ and $\pm 25\ nl/min$ for lower flow rates. Here, we characterise the uncertainty in the flow sensor within the range appropriate for mouse eye perfusion ($< 500\ nl/min$).

In order to evaluate the uncertainty in the flow sensors, we developed the following protocol. Firstly, using a bypass tube of large diameter, the pressure on the upstream and downstream ends of the flow sensor were set to be exactly equal, and the output from the sensor

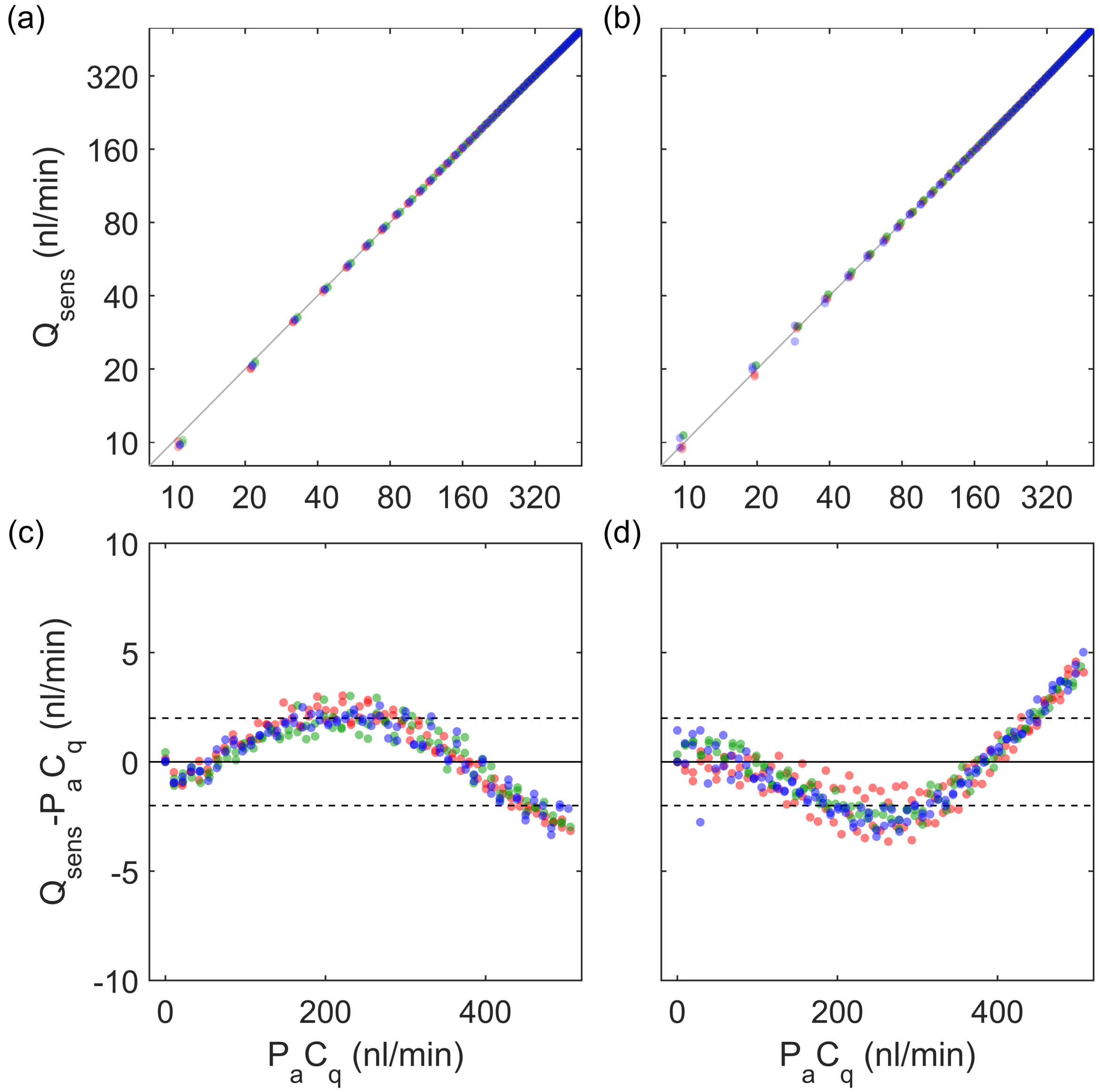

Figure S1-2: Analysis of the flow sensors. Results of uncertainty analysis of the flow sensor. The three colours indicate repeated measurements on separate days, including increasing and decreasing steps. (a) and (b) show the measured flow against the best estimate of the true flow (P_aC_q) for the two flow sensors used in the duplicate systems. (c) and (d) show the difference between the measured flow and P_aC_q. Dashed lines indicate the maximum uncertainty calculated according to Equation (S1-5).

was recorded over 20 seconds. The mean and variance over this period are defined as Q_{zero} and s^2_{zero}, respectively. The bypass tubing was then closed, and the upstream end of the flow sensor was opened to the actuated reservoir, whilst the downstream end was opened to an outlet reservoir at a fixed height. In the range of flow rates examined, there is negligible change in reservoir heights. The applied pressure, P_a, was then incremented by $0.1\ mmHg$ with 20

seconds at each step j. The central 10 seconds of each step are used to calculate the mean, Q_j, and variance, $s^2_{\text{Qave},j}$, of the flow rate. The applied pressure is increased in this manner until the flow rate exceeds $500 nl/min$, at which point the pressure is decremented until the flow rate is lower than zero.

A straight line fit with zero intercept was calculated for $Q_{\text{sens}} = Q_j - Q_{\text{zero}}$ against P_a using weighted linear regression, with the weights defined according to $\left(s^2_{\text{Qave},j} + s^2_{\text{zero}}\right)^{-1}$. The slope of this line is the hydrodynamic conductance of the flow sensor, C_q. As the true flow rate is unknown, it is estimated based on the applied pressure according to $Q_{\text{true}} = P_a C_q$, as C_q is theoretically constant. Figure S1-2 shows the results of these tests, with the colours indicating three tests on separate days. Figures S1-2a and b show the flow rate output from the sensor against $P_a C_q$, using a logarithmic abscissa to demonstrate the sensor accuracy at low flow rates. Figures S1-2c and d show the difference between the sensor output and the true flow rate, plotted against $P_a C_q$. The uncertainty in the flow sensor was calculated according to

$$s_{Q\text{sens}} = \sqrt{\frac{\sum_{i=1}^{N} \left(P_a C_q - Q_{\text{sens}}\right)^2}{N-1}} \tag{S1-5}$$

where N is the number of steps and $N-1$ is the number of degrees of freedom in the linear regression of Q_{sens} versus $P_a C_q$.

The average value of $s_{Q\text{sens}}$ from the three experiments on both sensors was $1.7\ nl/min$, whilst the maximum value was $2.0\ nl/min$. This maximum value is used as a conservative estimate of the standard deviation in flow rate attributable to uncertainty in the flow sensor and is indicated by the dotted lines in Figures S1-2c and d.

S2-4 Relative Uncertainty

Pressure Measurement Uncertainty

Despite the highly regulated environment, the measured pressure signal still retains a degree of uncertainty, predominantly due to electrical noise and minute movements of the tubing within the system. In order to define a steady state pressure, the pressure signal is averaged over a period of time, and the standard deviation of the pressure signal over this period gives a measure of uncertainty associated with the averaging. The data set of 66 individual eyes was analysed to calculate the values of $s_{P\text{ave}}$ arising from the averaging process. From the 490 pressure steps analysed, the median of $s_{P\text{ave}}$ was $0.009\ mmHg$ and the 95th percentile (used as a robust estimate of the upper limit) was $0.022\ mmHg$.

The total uncertainty in measured pressure for a given pressure step, j, can be estimated according to

$$s_{P,j}^2 = s_{P\text{ave}}^2 + s_{P\text{sens}}^2 \tag{S1-6}$$

Using the maximum sensor uncertainty and the 95th percentile on the uncertainty associated with averaging yields a conservative upper limit of $s_{P,j} = 0.025\ mmHg$. As the pressures recorded in the perfusion protocol range from approximately $4 - 20\ mmHg$, the maximum relative uncertainty in the pressure measurement is 0.6%.

Flow Measurement Uncertainty

An additional uncertainty arises due to averaging of the flow rate over the measurement period. Analysis of the 490 pressure steps from the 66 individual eyes yields a median $s_{Q\text{ave}}$ of $1.1\ nl/min$ with a 95th percentile of $2.3\ nl/min$.
The total uncertainty in the measured flow for a given pressure step j can be estimated as

$$s_{Q,j}^2 = s_{Q\text{ave}}^2 + s_{Q\text{sens}}^2 \tag{S1-7}$$

Based on the maximum sensor uncertainty and the 95th percentile averaging uncertainty, we get an upper limit of $s_{Q,j} = 3.1\ nl/min$. The flow rates measured in the mouse eye perfusions are dependent on the facility of a given eye, but considering a representative facility of $6nl/min/mmHg$ at the lowest perfusion pressure of $4mmHg$, the flow rate would be $24nl/min$. For this value, the uncertainty in the flow rate would be approximately 15% of the measured value.

We thereby conclude that the measurement uncertainty in the pressure is sufficiently small such that it can be neglected, whereas the uncertainty in the flow rate should be considered in the analysis.
