# Supplementary material for: Measurement of Outflow Facility Using iPerfusion
Source: PLoS One. 2016 Mar 7;11(3):e0150694. doi: 10.1371/journal.pone.0150694 (PMC4780770; doi:10.1371/journal.pone.0150694)
Supplement: S2 File — A description of the statistical methodology developed for use with iPerfusion, which takes into account the uncertainties in the measurements and includes the ‘weighted t-test’. (PDF) [file pone.0150694.s002.pdf]

Measurement of Outflow Facility using *iPerfusion*

Joseph M. Sherwood, Ester Reina-Torres, Jacques Bertrand, Barnaby Rowe, Darryl R. Overby

Supporting Information 2: Statistical Analysis

In this Supporting Information, we describe the statistical methodology developed for use with *iPerfusion*, which takes into account the uncertainties in the measurements and includes the 'weighted t-test'. MATLAB code for carrying out this analysis is can be provided upon request. We also provide a discussion of the reasons for our selection of approaches for reporting statistical results.

The sources of uncertainty involved in acquiring Y and s_Y^2 are described in the main text. Using these uncertainties, the average difference between the two populations, $\overline{Z}$, its variance, $s_{\overline{Z}}^2$, and the sample variance, s_Z^2, are evaluated. The number of degrees of freedom (DOF), ν, for $\overline{Z}$ is also calculated that along with the t-statistic enables calculation of a p-value relating to the null hypothesis, which in this case is considered to be no difference between samples. In addition, the confidence with which $\overline{Z}$ can be stated (confidence interval, CI) and the range of the treatment effect are evaluated.

Ultimately, the aim is to compare a 'control' population, A, to a 'treated' population, B. Depending on the experimental design, the data should be treated and interpreted in different ways. Here we consider three categories of experimental design involving two samples.

1. Unpaired data from different populations: such as comparing between two independent mouse strains or between different age groups of the same strain.

2. Unpaired data from a single population with treatment: for example, when analysing the effect of a systemically acting drug versus vehicle, using different individuals from the same population.

3. Paired data from a single population with treatment: for example, when analysing the effect of a drug that can be locally delivered to one eye, whilst the contralateral eye from the same individual serves as the vehicle treated control.

We start by describing the fundamental equations required for the statistical analysis, and then outline the analysis for each experimental design. Equations shown in blue represent the key relationships necessary for the final calculations.

Many of the techniques used in the following analysis require that the variable of interest be normally distributed. For parameters which are better described as lognormally distributed, such as outflow facility, we must carry out analysis on the log-transformed variable, $Y = \ln(C_r)$.

S2-1 Fundamental Equations

S2-1.1 Weighted Sample Statistics

Consider K values of a normally distributed parameter Z_k, with an uncertainty on each sample k of $s^2_{Z_k}$. The weights w_k can be defined according to

$$w_k = \frac{1}{s^2_{Z_k}} \tag{S2-1}$$

which can be normalised such that $\sum w'_k = 1$ according to

$$w'_k = \frac{w_k}{\sum\limits_{k=1}^{K} w_k} \tag{S2-2}$$

The weighted arithmetic mean is given by

$$\overline{Z} = \sum_{k=1}^{K} w'_k Z_k \tag{S2-3}$$

which has a weighted variance given by

$$s^2_{\overline{Z}} = \sum_{k=1}^{K} w'^2_k s^2_{Z_k} \tag{S2-4}$$

The unbiased weighted sample variance is given by

$$s^2_Z = \frac{\sum\limits_{k=1}^{K} w'_k \left(Z_k - \overline{Z}\right)^2}{1 - \sum\limits_{k=1}^{K} w'^2_k} \tag{S2-5}$$

For the special case where all samples are equally weighted, as occurs when each sample has an equivalent uncertainty, $s^2_{Z_k}$, the normalised weights equal to $1/K$. For this case, Equations S2-3, S2-4 and S2-5 become consistent with the typical definitions of the unweighted arithmetic mean, the square of the standard error of the mean and the unbiased variance, respectively.

S2-1.2 Welch-Satterthwaite Equation

When evaluating the CI and the p-value, it is necessary to consider the number of DOF associated with the measured value, given that at each stage of the analysis, we only have the sample variance, s, rather than exact values of the population variance, σ. For a normal paired t-test, the number of DOF is given by $\nu = K - 1$, whilst for an unpaired t-test with similar variances, $\nu = N_1 + N_2 - 2$, where N_1 and N_2 are the number of samples in the two populations. In the present approach, the weighting causes certain data points to have less influence than others,

and hence there are effectively fewer DOF. In order to estimate an appropriate value for ν, we use the Welch-Satterthwaite (W-S) equation. The W-S equation can be used to estimate the effective number of DOF for a variance, S^2, that can be written in the form of a linear combination of K independent sample variances, s_k^2, with with coefficients d_k, such that

$$S^2 = \sum_{k=1}^{K} d_k s_k^2 \tag{S2-6}$$

The W-S equation then calculates the number of DOF for S^2, ν_S, according to

$$\nu_S \approx \frac{\left(\sum\limits_{k=1}^{K} d_k s_k^2\right)^2}{\sum\limits_{k=1}^{K} \frac{\left(d_k s_k^2\right)^2}{\nu_k}} = \frac{\left(S^2\right)^2}{\sum\limits_{k=1}^{K} \frac{\left(d_k s_k^2\right)^2}{\nu_k}} \tag{S2-7}$$

where ν_k is the number of DOF in the calculation of each s_k^2.

S2-2 Unpaired Data from Independent Populations

In this case, there would be two populations with sample statistics $\overline{Y}_A,\ s_A^2$ and $\overline{Y}_B,\ s_B^2$. The difference between the weighted means of the two populations is given by

$$\overline{Z} = \overline{Y}_B - \overline{Y}_A \tag{S2-8}$$

We thus evaluate each population sample independently, and then combine the results.

S2-2.1 Calculating the Weights

We want to calculate the average value of Y for a sample of N eyes, $\overline{Y}$, as an estimate of μ_Y, the population mean. A simplified model for this can be written in terms of stochastic random variables, each assumed to be independent. For each eye, i, we can write

$$Y_i = \mu_Y + Y_{\text{pop},i} + Y_{\text{reg},i} \tag{S2-9}$$

where $Y_{\text{reg},i}$ is an error arising from uncertainty in the regression analysis, and $Y_{\text{pop},i}$ is the deviation in Y from μ_Y for eye i due to inherent variability within the population, both of which are assumed to have a zero mean. The variances can be written as

$$s_{Y_i}^2 = s_{\text{pop}}^2 + s_{\text{reg},i}^2 \tag{S2-10}$$

as μ_Y is an exact value and thus has no variance. Note that for the regression uncertainty, the variance is best characterised based on the known uncertainty for that eye, $s_{\text{reg},i}^2$, whereas

the variance in the population is best described by s^2_{pop}, calculated over the sample as follows. Applying Equation S2-10 over the sample using unweighted averaging yields

$$s^2_{\text{tot}} = s^2_{\text{pop}} + \overline{s^2_{\text{reg}}} \tag{S2-11}$$

where $\overline{s^2_{\text{reg}}}$ is the unweighted average measurement uncertainty and s^2_{tot} is the total unweighted variance in the measured values of Y_i given by

$$s^2_{\text{tot}} = \frac{1}{N-1}\sum_{i=1}^{N}\left(Y_i - \frac{1}{N}\sum_{i=1}^{N} Y_i\right)^2 \tag{S2-12}$$

Thus, an approximation of the population variance based on unweighted analysis is given by

$$s^2_{\text{pop}} \approx s^2_{\text{tot}} - \overline{s^2_{\text{reg}}} \tag{S2-13}$$

Equation S2-13 indicates that the variance in the population would be overestimated if we did not account for the measurement uncertainty, given that it is contributing to variability in the measured Y_i values. Substituting Equation S2-13 into Equation S2-10 yields

$$s^2_{Y_i} = s^2_{\text{tot}} + s^2_{\text{reg},i} - \overline{s^2_{\text{reg}}} \tag{S2-14}$$

According to Equation S2-14, the values of $s^2_{Y_i}$ will vary according to the relative difference of $s^2_{\text{reg},i}$ to the average $\overline{s^2_{\text{reg}}}$, and the effect of the weighting will decrease as s^2_{tot} increases relative to $\overline{s^2_{\text{reg}}}$. Finally, the weights are defined according to Equation S2-1 as

$$w_i = \frac{1}{s^2_{Y_i}} \tag{S2-15}$$

S2-2.2 Sample Statistics

The weighted arithmetic mean is calculated according to Equation S2-3

$$\overline{Y} = \sum_{i=1}^{N} w'_i Y_i \tag{S2-16}$$

and the variance of $\overline{Y}$ is given by Equation S2-4

$$s^2_{\overline{Y}} = \sum_{i=1}^{N} w'^2_i s^2_{Y_i} \tag{S2-17}$$

The unbiased weighted variance is given by Equation S2-5

$$s_{\overline{Y}}^2 = \frac{\sum_{i=1}^{N} w_i' \left(Y_i - \overline{Y}\right)^2}{1 - \sum_{i=1}^{N} w_i'^2} \tag{S2-18}$$

Figure S2-1 shows an overview of this analysis.

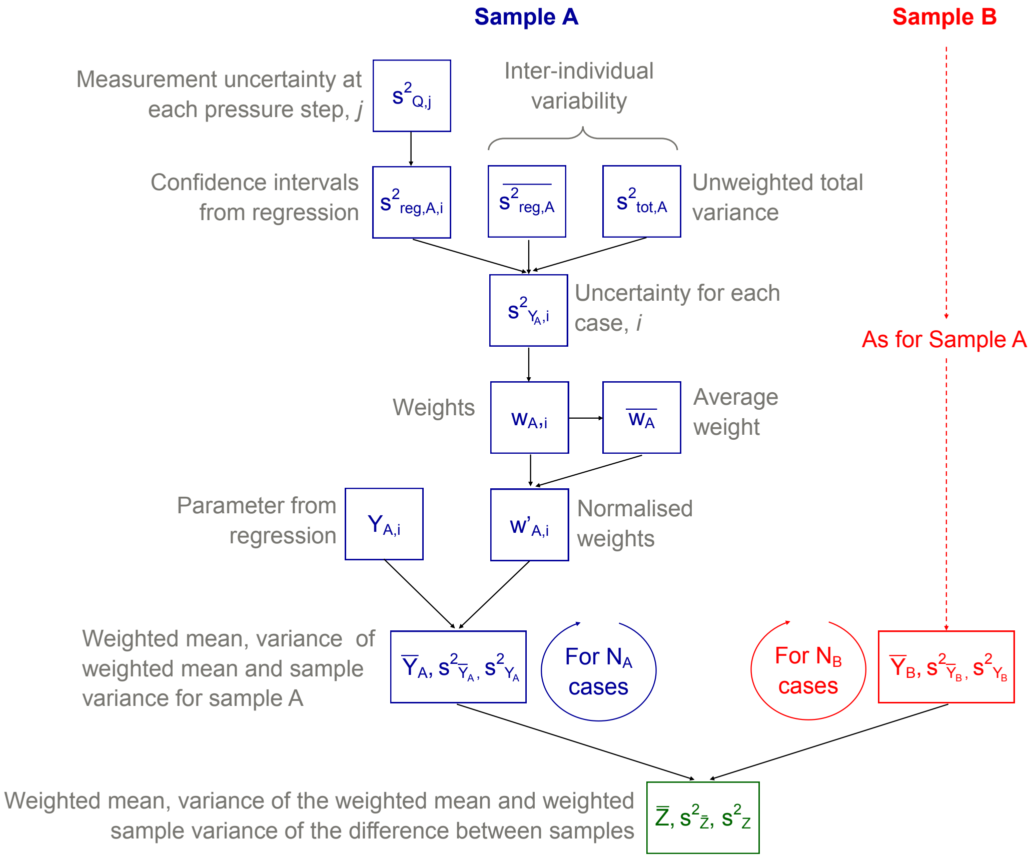

Figure S2-1: Schematic of the steps in the analysis method for unpaired data. Blue indicates control data, red indicates 'experimental' data and green indicates combined data on the differences between experimental and control cases.

S2-2.3 Degrees of Freedom

In order to calculate the CI for $\overline{Y}$ and a p-value corresponding to the null hypothesis that $\overline{Z} = 0$, we need to calculate $\nu_{\overline{Y}}$, the number of DOF for $\overline{Y}$. Combining Equations S2-17 and S2-14 yields

$$
\begin{aligned}
s_{\overline{Y}}^2 &= \sum_{i=1}^{N} w_i'^2 \left(s_{\text{tot}}^2 + s_{\text{reg},i}^2 - \overline{s_{\text{reg}}^2} \right) \\
&= s_{\text{tot}}^2 \sum_{i=1}^{N} w_i'^2 + \sum_{i=1}^{N} w_i'^2 s_{\text{reg},i}^2 - \overline{s_{\text{reg}}^2} \sum_{i=1}^{N} w_i'^2
\end{aligned}
\tag{S2-19}
$$

as s_{tot}^2 and $\overline{s_{\text{reg}}^2}$ are constant. Using the W-S equation (Equation S2-7) gives

$$
\nu_{\overline{Y}} \approx \frac{\left(s_{\overline{Y}}^2\right)^2}{\left(\frac{\left(s_{\text{tot}}^2\right)^2}{N-1} + \frac{\left(\overline{s_{\text{reg}}^2}\right)^2}{N} \right) \left(\sum_{i=1}^{N} w_i'^2 \right)^2 + \sum_{i=1}^{N} \frac{\left(w_i'^2 s_{\text{reg},i}^2\right)^2}{m_i - r}}
\tag{S2-20}
$$

where m_i is the number of data points in the regression fitting for each Y_i value and r is the number of free parameters in the model, in this case 2.

S2-2.4 Reporting Statistics for a Population

When reporting the value of $\overline{Y}$, it is preferable to also report its CI or margin of error (ME, defined as the half width of the CI), in order to establish how well the mean value is known. The $(1 - \alpha)\,100\%$ ME for the weighted mean of a given population is given by

$$
\text{ME}_{\overline{Y},(1-\alpha)} = s_{\overline{Y}}\, t_{\nu_{\overline{Y}},(1-\alpha/2)}
\tag{S2-21}
$$

where $t_{\nu_{\overline{Y}},(1-\alpha/2)}$ is the inverse of Student's t cumulative distribution function with $\nu_{\overline{Y}}$ degrees of freedom evaluated at $1 - \alpha/2$. As the sample size increases, $t_{\nu_{\overline{Y}},(1-\alpha/2)}$ will tend towards 1.96, the corresponding value for the normal distribution.

It is also beneficial to provide an indication of the range of values within the population. When using standard unweighted analysis, a multiple of the standard deviation of Y, $\sqrt{s_{\text{tot}}^2}$ would be reported to indicate the range of values within the population. However, as shown by Equation S2-11, this would be an overestimate as it would inherently include the additional uncertainty arising from the measurements. Using weighted analysis provides s_{Y}^2, an improved estimate of the sample variance as compared to the unweighted sample variance, s_{tot}^2. Thus, following the same logic as Equation S2-13, an improved estimate of the population variance is given by

$$
s_{\text{pop}}^2 = s_{Y}^2 - \overline{s_{\text{reg}}^2}
\tag{S2-22}
$$

We will report the spread in the population using $\pm 2 s_{\text{pop}}$, which is an indication of the interval within which 95% of facilities might be expected, which we refer to as two-sigma. For a given

population, we would therefore report the weighted mean and the 95% confidence interval on the weighted mean along with spread in the population:

$$\overline{Y} \pm \mathrm{ME}_{\overline{Y},95}\ (2 s_{\mathrm{pop}})$$

S2-2.5 Treatment Effect

Having defined $\overline{Y}_A$ for the N_A eyes in the control sample, $\overline{Y}_B$ for the N_B eyes in the treated sample and the corresponding measures of spread, the difference between the two populations can be analysed. The difference between the weighted sample means, $\overline{Z} = \overline{Y}_B - \overline{Y}_A$, is the best estimate of the difference between the average of the two populations. The variance in $\overline{Z}$ is given by

$$s^2_{\overline{Z}} = s^2_{\overline{Y}_B} + s^2_{\overline{Y}_A} \quad \text{(S2-23)}$$

The W-S equation (Equation S2-7) yields the number of degrees of freedom for $s^2_{\overline{Z}}$,

$$\nu_{\overline{Z}} \approx \frac{\left(s^2_{\overline{Z}}\right)^2}{\frac{\left(s^2_{\overline{Y}_B}\right)^2}{\nu_{\overline{Y}_B}} + \frac{\left(s^2_{\overline{Y}_A}\right)^2}{\nu_{\overline{Y}_A}}} \quad \text{(S2-24)}$$

where $\nu_{\overline{Y}_A}$ and $\nu_{\overline{Y}_B}$ are given by Equation S2-20. The ME for Z is then given by

$$\mathrm{ME}_{\overline{Z},(1-\alpha)} = s_{\overline{Z}}\, t_{\nu_{\overline{Z}},(1-\alpha/2)} \quad \text{(S2-25)}$$

In this context, the sum of the two independent populations variances

$$s^2_{\mathrm{pop},Z} = s^2_{\mathrm{pop},A} + s^2_{\mathrm{pop},B} \quad \text{(S2-26)}$$

would not have a useful interpretation. We would therefore not report the population variance of Z and only report $\overline{Z}$ and its 95% ME as

$$\overline{Z} \pm \mathrm{ME}_{\overline{Z},95}$$

S2-3 Unpaired Data from a Single Population with Treatment

Perfusion with a drug or other treatment would introduce an additional random variable into the analysis of the treated population, B, representing the variability in the effect of the treatment itself.

S2-3.1 Weights, Sample Statistics and Degrees of Freedom

The control population, A, would be analysed as described in Section S2-2. For population B, Equation S2-9 must be modified to give

$$Y_{B,i} = \mu_{Y_B} + Y_{\text{pop},B,i} + Y_{\text{reg},B,i} + Y_{\text{tre},i} \tag{S2-27}$$

where $Y_{\text{tre},i}$ is an additional deviation from μ_{Y_B} occurring due to the effect of the treatment. The variances are given by

$$s^2_{Y_{B,i}} = s^2_{\text{pop},B} + s^2_{\text{reg},B,i} + s^2_{\text{tre}} \tag{S2-28}$$

where the variance of the treatment effect is best described by s^2_{tre}. The unweighted average of Equation S2-28 across the sample is given by

$$s^2_{\text{tot},B} = s^2_{\text{pop},B} + \overline{s^2_{\text{reg},B}} + s^2_{\text{tre}} \tag{S2-29}$$

Thus, an approximation of the unweighted variance for population B is given by

$$s^2_{\text{pop},B} \approx s^2_{\text{tot},B} - \overline{s^2_{\text{reg},B}} - s^2_{\text{tre}} \tag{S2-30}$$

Combining Equation S2-28 and S2-30 yields

$$s^2_{Y_{B,i}} = s^2_{\text{tot},B} + s^2_{\text{reg},B,i} - \overline{s^2_{\text{reg},B}} \tag{S2-31}$$

which is identical to Equation S2-14 applied to population B because s^2_{tre} cannot be separated from $s^2_{\text{pop},B}$. Thus, the calculation of the weights, sample statistics and DOF for population B would follow the same approach used for population A (Equations S2-15 to S2-18 and S2-20).

S2-3.2 Treatment Variability

The unbiased weighted variance $s^2_{Y_B}$ (Equation S2-18) is an improved estimate of the total variance, relative to $s^2_{\text{tot},B}$. Thus we can rewrite Equation S2-29 according to

$$s^2_{Y_B} = s^2_{\text{pop},B} + \overline{s^2_{\text{reg},B}} + s^2_{\text{tre}} \tag{S2-32}$$

whilst the interpretation of $s^2_{Y_A}$ remains

$$s^2_{Y_A} = s^2_{\text{pop},A} + \overline{s^2_{\text{reg},A}} \tag{S2-33}$$

As both $s^2_{\text{pop},B}$ and $s^2_{\text{pop},A}$ are sample statistics describing the variability of the same population, we can make the assumption that $s^2_{\text{pop},B} = s^2_{\text{pop},A}$. By subtracting Equation S2-33 from S2-32, we can estimate s^2_{tre} as

$$s_{\text{tre}}^2 = s_{Y_B}^2 - s_{Y_A}^2 - \left(\overline{s_{\text{reg},B}^2} - \overline{s_{\text{reg},A}^2}\right) \tag{S2-34}$$

which is an estimate of the variability induced by the treatment. Thus, we would report our best estimate of the average treatment effect along with its 95% ME and the variability in the treatment effect as

$$\overline{Z} \pm \text{ME}_{\overline{Z},95}\,(2s_{\text{tre}})$$

S2-4 Paired Data

For paired data, rather than taking the difference between the weighted means from the two samples, we investigate the weighted mean of the differences Z_p for each pair, p, as given by

$$Z_p = Y_{p,2} - Y_{p,1} \tag{S2-35}$$

where $Y_{p,2}$ is the value of the treated sample from pair p, and $Y_{p,1}$ is the value of the vehicle treated sample from the paired (contralateral) control. $\overline{Z}$ is then the weighted mean of Z_p for Ψ pairs.

S2-4.1 Calculating the Weights

The difference between treated and control (vehicle-treated) samples of a given pair can be written in terms of stochastic random variables according to

$$Y_{p,2} + Y_{\text{reg},p,2} = Y_{p,1} + Y_{\text{reg},p,1} + \mu_Z + Z_{\text{tre},p} + Z_{\text{con},p} \tag{S2-36}$$

where $Y_{p,1}$ is the facility from the control eye from pair p, and $Y_{p,2}$ is from the corresponding treated eye. $Y_{\text{reg},p,1}$ and $Y_{\text{reg},p,2}$ are errors arising from the regression analysis. μ_Z is the population average of the paired differences arising due to the treatment. $Z_{\text{tre},p}$ is the deviation in the treatment effect from μ_Z for pair p. $Z_{\text{con},p}$ is the intra-individual variability that accounts for the difference in untreated baseline values between $Y_{p,1}$ and $Y_{p,2}$. All random variables except $Y_{p,1}$ and $Y_{p,2}$ have a zero mean, and μ_Z is an exact value. Combining Equations S2-35 and S2-36, yields

$$Z_p = Y_{p,2} - Y_{p,1} = \mu_Z + Z_{\text{tre},p} + Z_{\text{con},p} + Y_{\text{reg},p,1} - Y_{\text{reg},p,2} \tag{S2-37}$$

for which the variances are

$$s_{Z_p}^2 = s_{\text{tre}}^2 + s_{\text{con}}^2 + s_{\text{reg},p,1}^2 + s_{\text{reg},p,2}^2 \tag{S2-38}$$

Both s^2_{tre}, the variance in the treatment effect, and s^2_{con}, the variance between contralateral eyes, must be estimated by averaging over the entire sample, whereas the $s^2_{\text{reg},p}$ terms are known uncertainties from the regression analysis. Averaging Equation S2-38 across the sample,

$$s^2_{\text{tot},Z} = s^2_{\text{tre}} + s^2_{\text{con}} + 2\overline{s^2_{\text{reg}}} \tag{S2-39}$$

Substituting $s^2_{\text{tre}} + s^2_{\text{con}}$ into Equation S2-38 yields

$$s^2_{Z_p} = s^2_{\text{tot},Z} + s^2_{\text{reg},p,1} + s^2_{\text{reg},p,2} - 2\overline{s^2_{\text{reg}}} \tag{S2-40}$$

Note that the terms s^2_{con} and s^2_{tre} have cancelled. The implication of this is that the value of $s^2_{\text{tot},Z}$ encompasses the uncertainty in both the treatment effect between individuals and the intra-individual variability in untreated facility. Hence, if s^2_{tre} or s^2_{con} increased, we would observe an increase in $s^2_{\text{tot},Z}$. The weights can be defined according to

$$w_p = \frac{1}{s^2_{Z_p}} \tag{S2-41}$$

S2-4.2 Sample Statistics

The weighted mean according to Equation S2-3 is

$$\overline{Z} = \sum_{i=1}^{\Psi} w'_p Z_p \tag{S2-42}$$

for which the variance is given by Equation S2-4

$$s^2_{\overline{Z}} = \sum_{i=1}^{\Psi} w'^2_p s^2_{Z_p} \tag{S2-43}$$

The unbiased weighted variance is given by Equation S2-5

$$s^2_Z = \frac{\sum_{i=1}^{\Psi} w'_p \left(Z_p - \overline{Z}\right)^2}{1 - \sum_{i=1}^{\Psi} w'^2_p} \tag{S2-44}$$

Figure S2-2 shows an overview of this analysis.

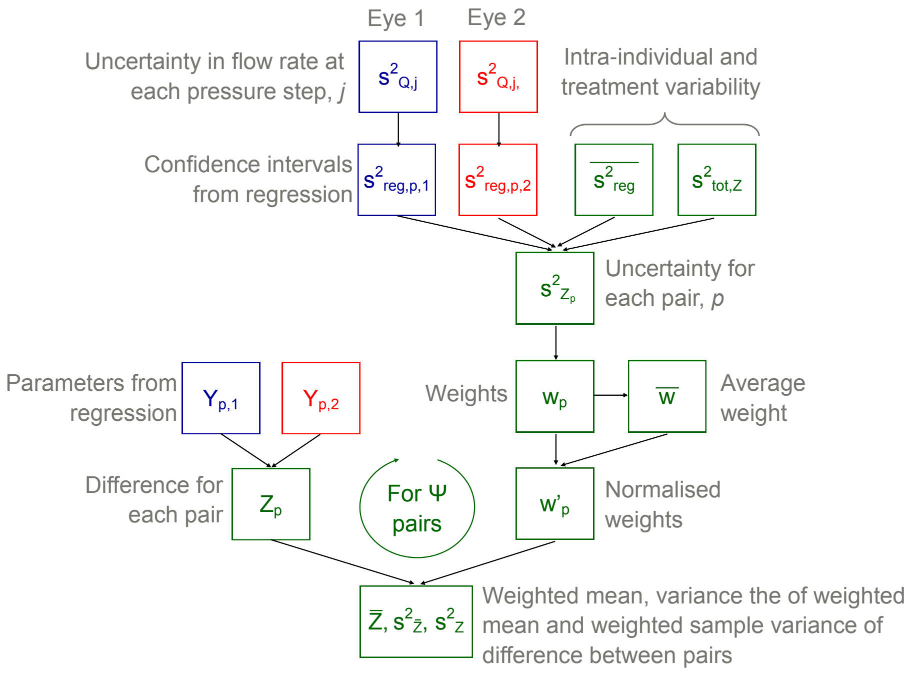

Figure S2-2: Schematic of the steps in the analysis method for paired data. Blue indicates control data, red indicates 'experimental' data and green indicates combined data on the differences between experimental and control cases.

S2-4.3 Degrees of Freedom

Equations S2-40 and S2-43 yield

$$\begin{aligned} s^2_{\overline{Z}} &= \sum_{p=1}^{\Psi} w'^2_p \left(s^2_{\text{tot},Z} + s^2_{\text{reg},p,1} + s^2_{\text{reg},p,2} - 2\overline{s^2_{\text{reg}}} \right) \\ &= s^2_{\text{tot},Z} \sum_{p=1}^{\Psi} w'^2_p + \sum_{p=1}^{\Psi} w'^2_p s^2_{\text{reg},p,1} + \sum_{p=1}^{\Psi} w'^2_p s^2_{\text{reg},p,2} - 2\overline{s^2_{\text{reg}}} \sum_{p=1}^{\Psi} w'^2_p \end{aligned} \tag{S2-45}$$

Using the W-S equation (Equation S2-7) gives

$$\nu_{\overline{Z}} \approx \frac{\left(s^2_{\overline{Z}}\right)^2}{\left(\frac{\left(s^2_{\text{tot},Z}\right)^2}{\Psi - 1} + \frac{2\left(\overline{s^2_{\text{reg}}}\right)^2}{\Psi} \right) \left(\sum_{p=1}^{\Psi} w'^2_p \right)^2 + \sum_{p=1}^{\Psi} \frac{\left(w'^2_p s^2_{\text{reg},p,1}\right)^2}{m_{p,1} - r} + \sum_{p=1}^{\Psi} \frac{\left(w'^2_p s^2_{\text{reg},p,2}\right)^2}{m_{p,2} - r}} \tag{S2-46}$$

S2-4.4 Treatment Effect

In standard unweighted analysis, the variance due to the treatment is typically approximated as the sample variance $s^2_{\text{tot},Z}$. However, as shown by Equation S2-39, $s^2_{\text{tot},Z}$ includes contributions from measurement uncertainty and intra-individual variability, and therefore $s^2_{\text{tot},Z}$ would tend to overestimate the true treatment variability. Given that the value of s^2_Z is an improved estimate of $s^2_{\text{tot},Z}$, we can rewrite Equation S2-39 as

$$s^2_{\text{tre}} = s^2_Z - s^2_{\text{con}} - 2\overline{s^2_{\text{reg}}} \tag{S2-47}$$

Where s^2_Z and $\overline{s^2_{\text{reg}}}$ are known. In order to estimate the intra-individual variability, s^2_{con}, and thus estimate s^2_{tre}, independent experiments must be carried out to measure the difference in Y values between untreated pairs, as described in the main text. As there is no treatment effect in this case, $\mu_Z = 0$ and $s^2_{\text{tre}} = 0$, and Equation S2-38 reduces to

$$s^2_{Z_p} = s^2_{\text{con}} + s^2_{\text{reg},p,2} + s^2_{\text{reg},p,1} \tag{S2-48}$$

Averaging Equation S2-48 over all pairs yields

$$s^2_{\text{dif}} = s^2_{\text{con}} + 2\overline{s^2_{\text{reg}}} \tag{S2-49}$$

where s^2_{dif} is the variance in the difference between Ψ_{con} untreated pairs, as given by

$$s^2_{\text{dif}} = \frac{1}{\Psi_{\text{con}}} \sum_{p=1}^{\Psi_{\text{con}}} Z_p^2 \tag{S2-50}$$

where the sample mean is defined as zero to be consistent with $\mu_Z = 0$. As $\overline{Z}$ for the untreated pairs is unlikely to be exactly zero, Equation S2-50 will yield a value of s^2_{dif} that is slightly larger than $s^2_{\text{tot},Z}$ for the control pair data set. We can therefore estimate s^2_{con} according to

$$s^2_{\text{con}} = s^2_{\text{dif}} - 2\overline{s^2_{\text{reg}}} \tag{S2-51}$$

The treatment variance s^2_{tre} is then given by Equation S2-47, and we report the mean weighted difference between pairs along with its 95% CI and spread of the data as

$$\overline{Z} \pm \text{ME}_{\overline{Z},95}\,(2s_{\text{tre}})$$

S2-5 Weighted *t*-test

The preceding analysis for either paired or unpaired data yields a value for $\overline{Z}$, $s^2_{\overline{Z}}$, and $\nu_{\overline{Z}}$. The variance of $\overline{Z}$ can be described as a χ^2 distribution with $\nu_{\overline{Z}}$ degrees of freedom, and thus the t-statistic given by

$$t = \frac{\overline{Z}}{s_{\overline{Z}}}$$

from which a p-value can be calculated based on a Student's t-distribution with $\nu_{\overline{Z}}$ degrees of freedom. This 'weighted *t*-test' provides an alternative to the standard *t*-test, by accounting for variable uncertainties in the measurements.

S2-6 Describing measures of spread

In this section, we discuss the reasons for the form proposed in the main paper for reporting statistical results. As described in the present study, for some variables, such as the outflow facility of mouse eyes, the assumption of normality may not be appropriate. However, it was found that a lognormal distribution is able to reasonably approximate the data, such that we can apply statistical methods dependent on the normal distribution to the log transform of the facility. In the interest of simplicity, we therefore discuss the implications of measures of spread for normally distributed data. Although the calculations used in the present study incorporate weighting in order to better account for uncertainties in the measurements, the following discussion also applies to non-weighted statistical descriptors.

S2-6.1 Selecting measures of spread for normally distributed variables

The standard deviation (SD) and the standard error on the mean (SEM) are the two most commonly used measures of spread for the reporting of outflow facility and other parameters in ocular biomechanics. These two parameters have specific and different meanings that should be considered. The SEM provides an indication of *the confidence on the estimate of the mean value*, whilst the SD describes *the spread of the data about the mean*.

Consider a study of a drug that may alter outflow facility. In this case, the SEM would inform how certain we can be about the average effect of the drug. The SEM is also used in hypothesis testing using the *t*-test, which estimates the probability that the average effect of the drug is in fact negligible (the null hypothesis). The SD would indicate the variability in the drug effect, which could be important if, for example, the drug was more or less effective in some patients. In practice, both parameters are useful, and in the interest of the most complete description of the data, we provide both the confidence on the mean and the spread within the population, in the form of functions of the SEM and SD.

S2-6.2 Defining measures of spread for normally distributed variables

Standard Deviation.
It should be noted that we only have s, the standard deviation of the sample, rather than σ, the true standard deviation of the population. s is therefore an estimate of σ.
The mean $\pm s$ (a single SD) describes the interval containing $\approx 68\%$ of the individual data points from a given sample. Rather than 68%, a range of 95% provides a more intuitive description of the spread of the data in the population. For this we use 'two-sigma', which in the case of the sample standard deviation is approximated by $2s$. The broad interpretation of mean $\pm$ two-sigma is thus that only 1 in 20 data points in the population sample would be expected to lie outside this range (rather than ≈ 1 in 3 for a single SD).
Standard Error on the Mean and Confidence Interval.
The mean $\pm$ SEM (a single SEM) broadly describes the range within which the mean lies with a probability of approximately 68%. Similarly to the SD, it is better to report a 95% probability as this provide a more intuitive range. Furthermore, we can calculate a CI, which has a more specific interpretation than two-sigma or equivalently, 2 SEM.
The strict definition of the 95% confidence interval is that if the experiment were repeated many times, sampling from the same population, the mean would lie within this range in 95% of the cases. More loosely, it can be interpreted as the range within which we can state that the mean probably lies.
If σ were known, then it would be possible to define the 95% confidence interval as 1.96 SEM, with the 1.96 (≈ 2) arising from the characteristics of the normal distribution. However, as only s is known, it is necessary to use the t-distribution to account for the reduced confidence on the estimate of the SD, arising from a small sample size. The t-distribution is similar to the normal distribution, but is more spread out, meaning that calculated probabilities are larger. The corollary is that we can calculate a value k, such that the mean $\pm$ kSEM describes the 95% confidence interval.
The value of k depends on the number of degrees of freedom (DOF) in the data, ν, defined as the number of data points minus the number of free parameters in the model. For unweighted paired data, the calculation of the mean has $\nu = \Psi - 1$ DOF (where Ψ is the number of pairs). For unpaired samples with similar sample variances, $\nu = N_A + N_B - 2$ (where N_A and N_B are number of data points in each population sample). For the weighted analysis in the present paper, the DOF is estimated as described above in this Supplemental Information. Table 1 lists the value of k for various DOF.

Table 1: Values of k for the 95% confidence interval, calculated from the inverse cumulative t-distribution

ν	3	4	5	6	7	8	9	10	20	50	100
k	3.18	2.78	2.57	2.45	2.36	2.31	2.26	2.23	2.09	2.01	1.98

As can be seen from Table 1, as ν increases, k decreases and thus the confidence interval becomes smaller. By reporting the mean, the confidence interval on the mean and two-sigma, the reader is provided with a more complete and intuitive description of the data, as compared to providing the mean with SD or SEM alone.
