# Supplementary material for: Measurement of Outflow Facility Using iPerfusion
Source: PLoS One. 2016 Mar 7;11(3):e0150694. doi: 10.1371/journal.pone.0150694 (PMC4780770; doi:10.1371/journal.pone.0150694)
Supplement: S3 File — Summary of the mathematical notation used in the main text. (PDF) [file pone.0150694.s003.pdf]

Measurement of Outflow Facility using *iPerfusion*

Joseph M. Sherwood, Ester Reina-Torres, Jacques Bertrand, Barnaby Rowe, Darryl R. Overby

Supporting Information 3: Nomenclature

This Supporting Information provides a list of all variables and parameters used in the main text. Table 1 defines the subscripts, Table 2 describes the main terms used in the paper, and Table 3 provides a list of sources of uncertainty and measures of spread.

S3-1 Subscripts

We use several subscripts to categorise variables, which are listed in Table 1.

Table 1: Subscripts

1	the control eye of a pair
2	the treated eye of a pair
A	the control population
B	the treated population
i	an index representing a given eye
j	an index representing a given pressure step
p	an index representing a given pair of eyes
r	reference: C_r is calculated at reference pressure P_r

S3-2 General nomenclature

Variables are listed in alphabetical order, with the exception of confidence intervals, CI, which follow the variable that they apply to.

Symbol	Units	Meaning
β	$-$	Non-linearity parameter in the power-law model for the flow-pressure and facility-pressure relationships (Equations 9 and 11): positive values indicate a pressure dependent increase in facility and negative values indicate a pressure dependent decrease
C	$nl/min/mmHg$	Total outflow facility, or simply 'facility', comprising both conventional outflow and any pressure-dependent components of unconventional outflow and AH secretion (pseudofacility), also used for the hydrodynamic conductance of the capillaries in the *in vitro* tests, as these values are analogous
C_{lin}	$nl/min/mmHg$	Facility when calculated assuming a linear Q-P relationship, implying a facility that is independent of pressure
$\overline{C}^{*}$	$nl/min/mmHg$	Weighted geometric mean of facility for a given population sample
$\text{ME}_{\overline{C}^{*},95}$	-	Margin of error on average facility
C_q	$nl/min/mmHg$	Hydrodynamic conductance of the flow sensor
C_r	$nl/min/mmHg$	Reference facility: the value of C at reference pressure P_r
D	mm	Internal diameter of inlet reservoir used in perfusion system
$\overline{D}^{*}$	$-$	Average fold change in facility
$\text{ME}_{\overline{D}^{*},95}$	-	Margin of error on fold change in facility
ΔP_q	$mmHg$	Pressure drop across flow sensor
g	m/s^2	Gravitational acceleration, $9.81\ m/s^2$
Γ	$nl/min/mmHg$	Instantaneous ratio of measured flow and pressure $\Gamma(t) = Q(t)/P(t)$: $\Gamma = C$ when the system reaches steady state
h_b	m	Height of the fluid surface in the inlet reservoir relative to that in the eye bath: see Figure 1a
h_r	m	Submersion depth of the eye: see Figure 1a
i	$-$	Index for a given eye
j	$-$	Index for a given pressure step
L_c	m	Length of glass capillary used for an *in vitro* test

m	kg	Mass of the fluid in the inlet reservoir
ME_{95}	-	Margin of error corresponding to the half-width of the 95% confidence interval
N	$-$	Number of eyes in an unpaired population sample
ν	$-$	Number of degrees of freedom
p	$-$	Index for a given pair of eyes
P	$mmHg$	Instantaneous pressure drop across the outflow pathway
P_a	$mmHg$	Applied pressure drop across the system, equal to $\rho g h_r$, related to P by Equation 5
P_e	$mmHg$	Pressure in the episcleral vessels
P_j	$mmHg$	Average pressure, P at steady state for a given pressure step
P_r	$mmHg$	Reference pressure: selected pressure at which to evaluate C_r
P_{I}, P_{II}	$mmHg$	Measured pressures at two pressure steps I and II, as used in the 'two-step' perfusion protocol (Equation 2)
Ψ	$-$	Number of pairs of eyes in a sample
Ψ_{con}	$-$	Number of pairs of eyes of untreated eyes for the estimation of s^2_{con}
Q	nl/min	Instantaneous flow rate into the eye from the perfusion system
Q_j	nl/min	Average flow rate at steady state for a given pressure step
Q_0	nl/min	Pressure independent outflow: the total outflow when the pressure drop across the pressure dependent pathways is zero
Q_{I}, Q_{II}	nl/min	Measured flow rates at two pressure steps I and II, as used in the 'two-step' perfusion protocol (Equation 2)
r	$-$	Pearson's product moment correlation coefficient
R_L	$mmHg/\mu l/min/mm$	Resistance per unit length of glass capillary used for *in vitro* testing
R_0	$mmHg/\mu l/min$	Resistance of the perfusion system downstream of the flow sensor
ρ	kg/m^3	Fluid density
Y	$-$	Log-transformed facility, $Y = \ln(C)$
$\overline{Y}$	$-$	Weighted mean log-transformed facility for a population sample
$\mathrm{ME}_{\overline{Y}^*,95}$	-	Margin of error on average log transformed facility

Z_p	–	Difference in log-transformed facility between paired eyes, for pair p
$\overline{Z}$	–	Weighted mean difference between treated and control eyes for a sample of paired eyes
$\mathrm{ME}_{\overline{Z},95}$	-	Margin of error on average change in log transformed facility

S3-3 Uncertainties and measures of spread

In this section we describe uncertainties and measures of spread (MOS). Uncertainties are defined as ambiguities or potential variabilities in the *measurements* due to error, noise or lack of certainty that affect the results and propagate throughout the analysis. MOS are defined as end-point descriptive statistics used to summarise the variability, predicted range or confidence for a *sample*.

A distinction should be made between 'arithmetic' or normal variables, and 'geometric' or lognormal variables. The arithmetic variables are all provided in the form s^2, indicating a variance, which allows addition in order to combine sources of uncertainty. For geometric variables, denoted with *, we provide the geometric standard deviations s^*. All values are dimensionless unless indicated.

Symbol	Applies to	Meaning
s^2_{con}	Paired eyes from a given population	Intra-individual uncertainty in Z: comprises tissue variability between paired eyes and uncertainty associated with interfacing the eye with the perfusion system
s^*_C	MOS: Sample of eyes	Geometric standard deviation in facility for a given population
$s^*_{\overline{C}}$	MOS: Sample of eyes	Geometric standard deviation on the geometric mean facility for a given population, could be interpreted as the standard error on the geometric mean
s^2_{dif}	A sample of paired eyes	Total variability in the differences in log-facilities for paired data: the unweighted variance of Z_p
$s^*_{\overline{D}}$	MOS: Paired/Unpaired comparison	Geometric standard deviation of fold change between two populations
$s^2_{Q\text{ave}}$	Pressure step, j	Uncertainty in flow rate due to averaging a noisy signal, units: $(nl/min)^2$
$s^2_{Q\text{sens}}$	A given flow ensor	Uncertainty in flow rate due to sensor uncertainty, units: $(nl/min)^2$
$s^2_{Q,j}$	Pressure step, j	Uncertainty in flow rate for a given pressure step j, due to averaging a noisy signal and sensor uncertainty, units: $(nl/min)^2$
$s^2_{P,j}$	Pressure step, j	Uncertainty in pressure for a given pressure step j, due to averaging a noisy signal and sensor uncertainty, negligibly small for the present system, units: $mmHg^2$

s^2_{pop}	A sample of unpaired eyes	Inter-individual uncertainty in Y: comprises tissue variability between unpaired eyes from the same population and uncertainty associated with interfacing the eye with the perfusion system
$s^2_{\text{reg},i}$	A single eye, i	Uncertainty in log facility for a given eye, i, arising from regression fitting of model to $Q-P$ data, assumed to be equal to the 68% confidence interval on Y
$\overline{s^2_{\text{reg}}}$	A sample of eyes	Unweighted mean of $s^2_{\text{reg},i}$ for a given sample
s^2_{tot}	A sample of unpaired eyes	Total unweighted variance in log-facility for a given population sample: the unweighted variance of Y_i
$s^2_{\text{tot},Z}$	A sample of paired eyes	Total unweighted variance in difference in log-facility for a sample of paired eyes: the unweighted variance of Z_p
s^2_{tre}	Sample of treated paired or unpaired eyes	Additional uncertainty in Z arising from variable effects of the treatment
s^*_{tre}	MOS: Paired/Unpaired comparison	Geometric standard deviation of fold change due to treatment variability
s^2_Z	MOS: Paired/Unpaired comparison in log domain	Unbiased weighted variance of Z
$s^2_{\overline{Z}}$	MOS: Paired/Unpaired comparison in log domain	Unbiased variance of $\overline{Z}$, qualitatively comparable to the square of the standard error on the weighted mean of Z
s^2_Y	MOS: Sample of unpaired eyes	Unbiased weighted variance of Y
$s^2_{\overline{Y}}$	MOS: Sample of unpaired eyes	Unbiased variance of $\overline{Y}$, qualitatively comparable to the square of the standard error on the weighted mean of Y
